# Empirical Mode Decomposition and k-Nearest Embedding Vectors for Timely Analyses of Antibiotic Resistance Trends

**DOI:** 10.1371/journal.pone.0061180

**Published:** 2013-04-25

**Authors:** Douglas Teodoro, Christian Lovis

**Affiliations:** Division of Medical Information Sciences, University Hospitals of Geneva, Geneva, Switzerland; Amphia Ziekenhuis, Netherlands

## Abstract

**Background:**

Antibiotic resistance is a major worldwide public health concern. In clinical settings, timely antibiotic resistance information is key for care providers as it allows appropriate targeted treatment or improved empirical treatment when the specific results of the patient are not yet available.

**Objective:**

To improve antibiotic resistance trend analysis algorithms by building a novel, fully data-driven forecasting method from the combination of trend extraction and machine learning models for enhanced biosurveillance systems.

**Methods:**

We investigate a robust model for extraction and forecasting of antibiotic resistance trends using a decade of microbiology data. Our method consists of breaking down the resistance time series into independent oscillatory components via the empirical mode decomposition technique. The resulting waveforms describing intrinsic resistance trends serve as the input for the forecasting algorithm. The algorithm applies the delay coordinate embedding theorem together with the k-nearest neighbor framework to project mappings from past events into the future dimension and estimate the resistance levels.

**Results:**

The algorithms that decompose the resistance time series and filter out high frequency components showed statistically significant performance improvements in comparison with a benchmark random walk model. We present further qualitative use-cases of antibiotic resistance trend extraction, where empirical mode decomposition was applied to highlight the specificities of the resistance trends.

**Conclusion:**

The decomposition of the raw signal was found not only to yield valuable insight into the resistance evolution, but also to produce novel models of resistance forecasters with boosted prediction performance, which could be utilized as a complementary method in the analysis of antibiotic resistance trends.

## Introduction

Antibiotic resistance is a major public health problem, leading to excess morbidity and mortality rates worldwide [1]. As an example of its impact, the World Health Organization chose combating antimicrobial resistance as the theme of the World Health Day 2011. The lack of on-demand evidence to support antibiotic prescribing and the consequent misuse of antibiotics are regarded widely as some of the underlying causes of increasing antibiotic resistance [2]. Phenotype-based antimicrobial susceptibility tests typically take two to three days to provide results on the pathogen’s susceptibility. As a result, clinicians have often to prescribe a treatment empirically without the ultimate evidence on the agent’s effectivity [3]. Another factor associated to the further spread of resistance is the delay in spotting emerging trends and resistance outbreaks in current biosurveillance systems [4], [5]. The use of yearly aggregated data, while crucial to assess the efficacy of interventions and plan future public health policies [6], does not bring sufficient reactivity to impact clinical practice in a timely manner. It reduces the ability to detect readily the appearance of new resistance clones and, consequently, to control their spread in hospitals and in the community.

At the point of care, short- (days, weeks) and medium-term (months) resistance trend analyses using up-to-date microbiology data are essential for care providers and infection control groups because these types of analyses depict more faithfully the current dynamics within the clinical setting, especially in units with high selective pressure [3]. For example, up-to-date resistance data are a valuable source of information for clinical decision support systems, such as those supporting empirical therapy [7] but also to create guidelines for bacterial treatment and hospital infection control policies. As demonstrated in [8], the injection of resistance information in automatic guideline generation systems significantly improves the precision of selecting the most appropriate antibiotic for a given treatment condition. Moreover, accurate predictive models for short-term resistance trends are important components of outbreak detection biosurveillance systems, serving as a reference value between an endemic and pandemic judgment [9].

Whereas long-term (years) trends are relatively easy to extract and forecast due to the slow dynamics in the evolutionary timescale [10], medium- and, particularly, short-term resistance time series are more challenging to model. Several ecological models have been developed to describe how resistance varies over time. They were primarily used to assess the impact of interventions on resistance [11], [12]. However, by estimating optimally the parameters employed, they could also serve to predict the evolution of short- and medium-term trends [13]. Despite their potential value to support the design of infection control and intervention programs [14], these models have some drawbacks as a general antibiotic resistance forecasting approach. First, some of them are theoretically valid only under certain a priori conditions, such as constant antibiotic pressure, which are often violated in actual clinical practice [15]. Second, they incorporate several biological and clinical variables, which are in many cases difficult to estimate accurately. For example, the value of the parameter associated with the transmission rate, which is key to these models, is notoriously difficult to estimate precisely [16]. Finally, some models, like those based on logistic regression [13], may fail in trivial cases where resistance starts to decrease over time after some initial increasing – a phenomenon verified often from actual microbiology data [17].

Taking into account the model-driven approaches above and their known weaknesses, we investigate the use of data-driven methods to provide timely resistance trend forecasting for enhanced biosurveillance systems. Our approach combines robust trend extraction and prediction methods that do not make any a priori assumptions of the underlying bacterial and antibiotic resistance dynamics. Therefore, it can be applied in different contexts, independent of the agent at stake. Our methodology operates by extracting waveforms that describe different variation modes of resistance time series. This is performed using the empirical mode decomposition (EMD) algorithm [18], an adaptive and data-driven technique that represents the signal as a set of oscillatory functions plus a monotonic residual that accounts for the intrinsic resistance trend. Further, the decomposed waveform components are used as the input to a machine learning algorithm supported by the k-nearest neighbor (k-NN) method [19] to provide the resistance forecasts. Instead of deploying the full signal spectrum in the learning process, we select only components that contribute significantly to the underlying signal, that is, those that differ from noise. The learning algorithm uses the delay coordinate embedding technique [20], [21] to capture the dynamics of the different time series. Then, using the mean output of k-nearest embedding vectors, it projects observed resistance events in the future dimension. The model is trained and tested using leave-one-out cross-validation in a large data set of resistance time series spanning a decade of antibiotic susceptibility tests. We demonstrate some trend extraction analysis use-cases and compare the accuracy of our forecasting model with other machine learning forecasting approaches.

## Methods

In this study, we use retrospective time series of anonymized and weekly aggregated antibiograms to develop and test a model for extraction and prediction of antibiotic resistance trends applied to up-to-date and short-term resistance changes. The data set was provided by the microbiology laboratory of the University Hospitals of Geneva (HUG). HUG is a consortium of all public and teaching health facilities of the canton of Geneva, Switzerland. Permission to use anonymized population aggregated information was granted through the Detecting and Eliminating Bacteria Using Information Technology (DebugIT) large scale integration project [22], within which HUG collaborated as a data provider. The data were extracted using ARTEMIS [23]–[25], a transnational antimicrobial resistance monitoring system developed within DebugIT.

The statistics of the data set used to train and assess the system are presented in Table 1. The training set contains twenty six resistance time series of four main pathogens – *Escherichia coli, Klebsiella pneumonia, Pseudomonas aeruginosa* and *Staphylococcus aureus* – tested against a set of antibiotics, selected according to their relevance in susceptibility tests and antibiotherapies. Each time series comprises a decade of resistance information, containing weekly data from January 1, 2002 to December 31, 2011 in 520 data points. The forecasting algorithm was trained using leave-one-out cross-validation adapted to time series forecasting, where past events are held in the training set and only future data points are left out for testing. The minimum number of observations necessary to train the system was set to 350 weeks, resulting in a test set of 170 data points (see Figure S1 for excerpt of the time series covering the testing period). In the following sections, we describe the trend extraction and forecasting methods in details.

**Table 1:** Weekly resistance rate time series **–** means and standard deviations (SD).

Time series	Organism	Antibiotic	Mean (%)	SD
EC 1	*E. coli*	AMI^1^	6.42	2.91
EC 2	*E. coli*	AMP^2^	47.22	7.39
EC 3	*E. coli*	AMC^3^	12.96	6.24
EC 4	*E. coli*	FEP^4^	6.59	5.73
EC 5	*E. coli*	CF3^5^	6.92	5.75
EC 6	*E. coli*	FLU^6^	16.76	6.25
EC 7	*E. coli*	SXT^7^	27.47	6.07
KP 1	*K. pneumonia*	AMI	7.32	7.42
KP 2	*K. pneumonia*	AMC	14.03	11.82
KP 3	*K. pneumonia*	FEP	10.80	10.59
KP 4	*K. pneumonia*	CF3	10.91	10.63
KP 5	*K. pneumonia*	FLU	9.07	9.07
KP 6	*K. pneumonia*	TZP^8^	3.95	5.90
KP 7	*K. pneumonia*	SXT	17.26	12.18
PA 1	*P. aeruginosa*	AMI	7.11	4.99
PA 2	*P. aeruginosa*	CAR^9^	11.34	6.54
PA 3	*P. aeruginosa*	FEP	3.94	4.05
PA 4	*P. aeruginosa*	CAZ^10^	6.52	4.97
PA 5	*P. aeruginosa*	CIP^11^	8.14	5.96
PA 6	*P. aeruginosa*	TZP	6.79	9.36
SA 1	*S. aureus*	AMI	33.40	13.06
SA 2	*S. aureus*	PEN^12^	92.20	5.32
SA 3	*S. aureus*	CLI^13^	38.28	10.08
SA 4	*S. aureus*	FLU	39.10	12.30
SA 5	*S. aureus*	MAC^14^	41.66	10.29
SA 6	*S. aureus*	SXT	1.56	1.83

Time series of weekly resistance rates defined as the percentage (%) of resistant tests from the total of antibiograms (including intermediate results) for four groups of pathogens **–** *Escherichia coli*, *Klebsiella pneumonia*, *Pseudomonas aeruginosa* and *Staphylococcus aureus*.

1 aminoglycoside;

2 aminopenicillin;

3 amoxicillin-clavulanic acid;

4 cefepime;

5 3rd generation cephalosporin;

6 fluoroquinolone;

7 trimethoprim-sulfamethoxazole;

8 piperacillin-tazobactam;

9 carbapenem;

10 ceftazidime;

11 ciprofloxacin;

12 benzylpenicillin;

13 clindamycin;

14 macrolide.

### Extraction of Resistance Trends

We use the EMD algorithm to decompose and extract trends of the resistance rate time series. EMD is an empirical, adaptive and fully data-driven method for signal decomposition suitable for nonlinear and non-stationary processes [18], [26], [27]. Contrary to other signal decomposition methods, such as Fourier transformation and wavelets, which assume arbitrary functions for the underlying process (sinusoid and wavelet basis functions respectively), EMD is adaptive to the natural scales of the data and thus can reveal the features of the time series more precisely [28]. The method works by breaking down the signal as superpositions of local intrinsic modes of oscillation called Intrinsic Mode Functions (IMFs). According to Huang *et al.* [18], each IMF satisfies two particular conditions: (*i*) in the whole data set, the number of extrema, that is, the local minima or maxima, and the number of zero crossings must either equal or differ at most by one; and (*ii*) at any point, the mean value of the envelopes defined by the local maxima (upper) and the local minima (lower) is zero. The result of the EMD algorithm is a set of IMF components, with zero mean and unrestricted amplitude and frequency along the time axis, and a residual component, which accounts for the mean underlying trend.

**Figure 1:**
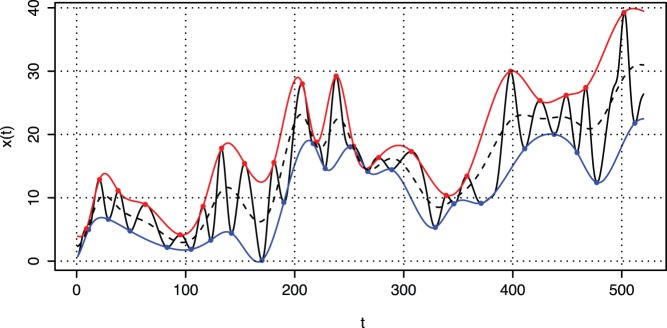
EMD –IMF sifting process. Signal 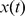 **–** black continuous line; upper envelope 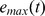 **–** red line; lower envelop 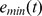 **–** blue line; local mean 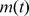 **–** black dashed line.

IMFs are extracted from the signal through a sifting process, which can be implemented according to the following algorithm (see Figure 1):

1. Identify the local maxima and minima of a signal 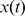.
2. Connect the local maxima with a cubic spline as the upper envelope 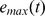. Repeat the process for the local minima to create the lower envelope 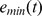.
3. At every time point 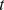, calculate the local mean 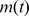 given by the average of the upper and lower envelopes: 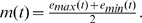
(1)
4. Obtain the first oscillatory component 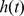 by taking the difference between the data signal 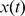 and the local mean 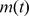, that is, 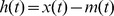.
5. If 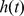 is not an IMF, it is taken as the new signal 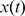 and steps 1–4 are repeated until 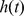 satisfies the IMF conditions. The final 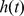 is designated as 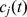, the *j*th IMF component.
6. Once an IMF component has been identified, it is subtracted from the signal, leaving a residual 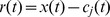. Steps 1–5 are repeated with 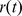 taking the place of 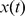 until 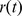 becomes a monotonic function from which no more IMFs may be extracted.

After the data signal 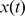 has passed through the IMF sifting process, it can be represented in terms of the IMFs 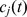 and the monotonic residual component 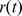 as
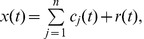
(2) where 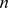 is the number of IMFs obtained in the sifting process.

The residual 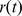 in Equation 2 provides the mean trend of the signal. According to Huang *et al.* [18], the oscillatory components 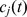 are orthogonal functions and may represent physically meaningful signals, which in the case of resistance time series could theoretically express short-, medium- and long-term trends and eventually outbreaks. The first component 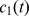 has the smallest time scale and thus corresponds to the highest frequency component. As such, it is associated to noise. Notice that the components are extracted using a fully data-driven process, where it is not required to predetermine any basis functions. Therefore, this methodology is adaptive to any time varying signal, which makes it suitable to extract trends from the different bacterial-antibiotic resistance time series.

### Forecasting Resistance Trends

#### Delay coordinate embedding

The theoretical background of our forecasting algorithm is derived from the delay coordinate embedding theorem [20], [21]. This theorem describes a phase space reconstruction technique that provides the conditions for nonlinear dynamic systems to be reconstructed from a finite sequence of observations of the system’s state. Let us consider a time series 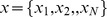 represented by a sequence of 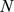 observations generated from a nonlinear function. In delay coordinate embedding, vectors in the new phase space, the embedding space, are defined by
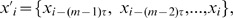
(3)where 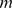 is the embedding dimension and 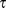 is the delay time or lag relative to the sampling rate. Equation 3 provides a multidimensional representation of a unidimensional nonlinear time series, which according to Takens [20] and Sauer *et al.* [21] can reconstruct the observations made with a generic unknown function 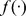 of a nonlinear dynamical system. The dimension 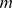 can be considered as the minimum number of state variables required to describe the system. For the sake of exposition, in the remainder of the paper we take by convention 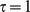.

Now, considering the same one-dimensional time series 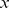 generated by a system 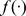, whose values we are trying to predict, a standard approach in time series forecasting is to project future values using a function of past observations such that
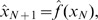
(4) where 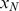 is current system state, 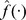 is the estimated function of the actual system 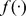 and 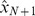 is the estimate of the next system state. Similarly, we can project the future system state using the delay vectors of Equation 3 such that 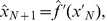
(5)where 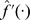 is the estimated function in the embedded space of the actual system 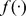. Under suitable assumptions on the dynamics, the correspondence presented in Equation 5 is one-to-one, which means that the behavior of the nonlinear system is accounted for in the behavior of the delay coordinate embedding defined by the mapping 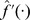 [20].

There are several ways to estimate the function 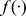 for obtaining the point forecast 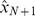 [29]. Our model employs the k-NN framework as a piecewise estimator of 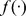. The k-NN algorithm implements a function approximator 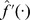 that stores a set of mappings 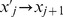, with 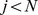, where the delay coordinate vector 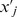 acts as a surrogate for 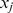. When the algorithm is tested against a point forecasting query 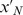, the 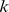 delay vectors having the shortest Euclidean distance to the query state are extracted and the mapping outputs 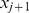 are used to obtain 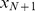. If 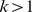, the value of the point forecast is computed as the average of the 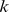 extracted samples, that is,
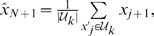
(6)where 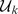 is a neighborhood of size 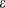 in the space defined by the embedding vectors 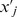 and 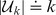 is the number of neighbors.

#### The k-nearest embedding vector forecasting algorithm

**Figure 2:**
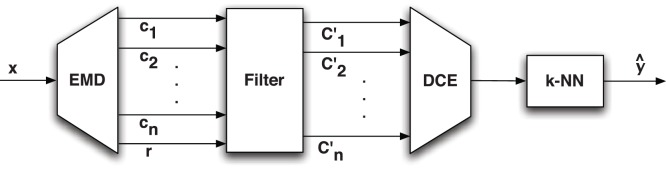
High-level block diagram of the k-nearest embedding vectors forecaster. The *empirical mode decomposition* (*EMD*) block decomposes the input 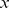. Then, the *filter* block selects the time series functions 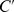 that are relevant to the signal. Further, the *delay coordinate embedding* (*DCE*) block determines the embedding dimension 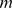 and embeds the signal 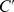 into a multidimensional space. Finally, the *k-nearest neighbor* (*k-NN*) block calculates the distance between the input query and the training points in the embedded space and makes the projection in the future dimension to obtain the forecast 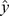.

Our forecasting system is depicted in Figure 2. To simplify discussion, we refer from now on to the IMF components 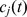 and the residue 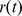 obtained from the decomposition process simply as *components*, and denote them by the vector 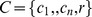 of length 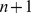, where 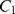 corresponds to the first IMF and 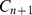 to the residue 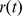. The system breaks down the input resistance time series 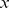 into several oscillatory components using the EMD algorithm. Then, components that do not contribute to the signal are removed and those remaining are embedded into delay vectors of dimension 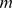. Further, the algorithm is trained to compute the size of the delay vector neighborhood 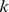, which will provide the best estimate of the forecast 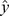. Thus, from a machine learning viewpoint, the tasks of the learning algorithm sum up, firstly, to identify the EMD components 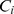 that represent best the system being modeled, secondly, to estimate the dimension 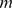 of the embedding sequences and, finally, to compute the number 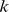 of nearest neighbors that encompass the dynamics of the system.

In the following steps, we summarize the computationally efficient leave-one-out cross-validation algorithm that we used to train and test the forecasting system:

1. Divide the input time series of size 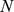 into two independent parts: a training set 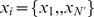 and a testing set 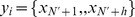, where 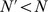 is the minimum number of observations necessary to fit the model and 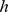 is the forecasting horizon.
2. Decompose the training time series 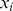 into components 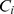 using the EMD algorithm.
3. Select the subset of components 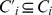 that are relevant to the learning model (this is described below).
4. Compute the optimal embedding dimension 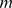 for the space created by 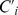 (this is described below).
5. Embed the components 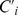 into a space of dimension 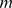 and together with the respective one-step-ahead output create the training set 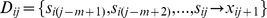, where 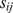 is a vector containing the *j*th elements of the components 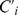 and 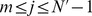. Then, compute the optimal number of nearest neighbors 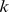 for the training set 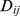 using cross-validation.
6. For 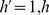:
  - Create the test input 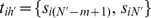 from the last embedded vector obtained from components 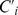. Then, using the training model 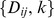, find the 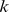 nearest neighbors of the test input 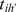. Finally, project the 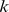 embedding vectors into the dimension 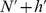 using Equation 6 for estimating the 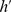-step-ahead forecast 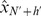.
  - While 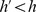, concatenate the forecast outcome 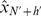 into the time series 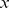 and repeat steps 2 and 3 to update the components 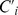.
7. Compute the residual error using the forecasted values 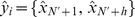: 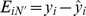.
8. Increase 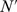 and go to step 1 while 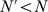.
9. Once each time point in the test set has been predicted, compute the overall cross-validation mean absolute error 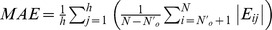 and root mean squared error 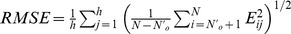, where 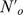 is the initial minimum number of observations.

In our algorithm, predictions are made using the latest resistance time points. Horizons greater than one are estimated taking the one-step-ahead forecast as the last time point. An alternative algorithm would create a training set for each h-step-ahead of interest and, at step 5, the one-step-ahead mapping would be replaced by a h-step-ahead. Then, the loop in step 6 would be avoided. However, it would require one training model for each step-ahead forecast 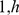, which is computationally more expensive.

#### Selecting the components *C*′

**Figure 3:**
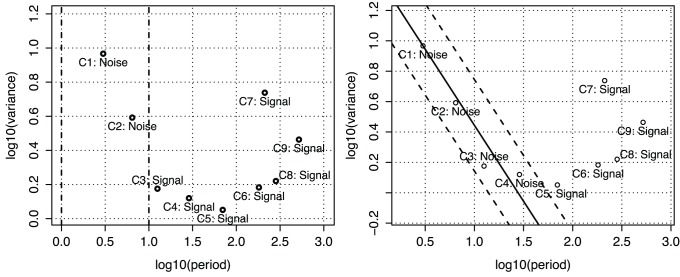
Component selection models. A component labeled as *noise* implies that it is not distinguishable from a pure white noise series. Thus, it cannot be learned by the machine learning algorithm. *Left -* example of the empirical selection model using a threshold filter (*DECF*), where components with period smaller than 10 weeks (or 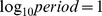) are excluded from the learning algorithm. *Right -* example of the component selection model using the Wu and Huang [26] expectation of variance approach to define statistically significant components (*DECS*).

We envisage three models to select the relevant EMD components 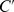. The first model, *DECA*, does not actually filter any component and thus the system is trained with the full signal spectrum. For the other two models, we make a fair assumption that the machine learning algorithm cannot learn the noisy components and hence they shall be excluded from the model to avoid a negative impact on the forecasting. The remaining components, which correspond to the physically meaningful signals, are then used to train the system. Based on this assumption, the second model (see Figure 3 - left), *DECF*, filters out noisy components using a frequency threshold. High frequency components are empirically associated with noise. We consider a period of 10 weeks as the minimum necessary to learn the signal. Components with shorter cycles are filtered out. The last model (Figure 3 - right) uses a statistical significance test derived by Wu and Huang [18], [26] to distinguish between noise and signal in the IMF components. The test assumes that the first IMF is a random noise. Then, other components are compared with this IMF using a distance metric proportional to the logarithms of the component’s variance and period [26]. The components whose variance and period exceed the noisy boundaries are considered to contain statistically significant information for the resistance trends. In our experiments, we use a 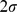 distance for the noisy boundaries. This model is further referred as *DECS*.

#### Determining the embedding dimension *m*

We propose two methods to determine the dimension 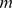 of the delay vectors. In the first approach, we naïvely set the embedding dimension to a fixed size. In the second, we use a modified version of a methodology derived in [30] that applies fractal dimensions to specify dynamically the optimal length of the delay vectors. According to the authors, the fractal dimension 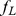 of the time series, which gives the intrinsic dimensionality of the embedding vectors in the embedding space, can determine the optimal value of the embedding dimension 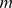. In their algorithm, 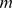 is incremented between the range 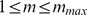 and 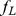 is calculated for each space 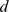 created. After some value of 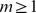, increasing the embedding dimension does not add any relevant information regarding the state space, which is verified by a flattening in the slope of 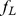. The turning point, which lies within 95% of the maximum 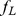, is taken as the optimal 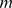. Further details of the algorithm can be found in [30]. Since we have several components, we calculate 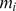 for each space defined by the components 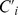 and the final 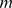 is defined as the mean of 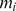. We use time series *EC 4*, *KP 2*, *PA 5* and *SA 5* and the *DECF* model to train the best method, and consequently the dimension 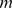. The naïve approach is trained for 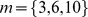 and 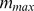 is set to 10 in the fractal dimension method.

### Performance Measures

The results of the trend extraction method are provided using two use-cases of resistance trend analysis. Due to the lack of standard and formal definitions for trend, there is no benchmark for trend extraction. Thus, it is difficult to quantitatively measure trend extraction methods. To demonstrate qualitatively the power of the EMD algorithm, we first present the statistics on the oscillatory period of the resistance components. Second, we correlate components from the resistance time series with components of time series that may be associated with resistance. We take a temperature time series from the Geneva region as an example.

For the machine learning forecaster, we provide the results for 1, 3 and 12 week-ahead forecasting horizons using the MAE and RMSE cost functions. Since MAE and RMSE measure the deviation between actual and predicted values, the smaller the values of MAE and RMSE the closer the predicted time series is to the true time series. Results of the models *DECA*, *DECF* and *DECS* are compared to a baseline approach based on the random walk method, which is the standard benchmark in machine learning forecasting [31], and to a k-NN regression applied to the raw signal with 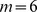.

### Statistical Analysis

We use R version 2.15.0 to decompose the resistance trends, implement the machine learning models and perform the statistical analyses. We apply the paired two-sided Wilcoxon test to compare the error of the forecasting models. *P*-values lower than.05 are considered significant. Correlation statistics are reported using the Pearson’s coefficient of correlation. Supplementary information on the software implemented is available from the authors upon request.

## Results

In the following sections, we present the results of the trend extraction and forecasting methods for timely analyses of antibiotic resistance trends. Because this study yielded several hundred results, we provide aggregated statistics and some prominent examples for each of the evaluation dimensions. The full result set is provided as supplementary material. We start by displaying some qualitative analyses of the trend extraction methods, where the EMD technique is used to extract periodicity of the time series and to correlate resistance trends with external factors that may be associated with changes in resistance. Then, we present the results of the machine learning forecasting, where the performance in terms of MAE and RMSE metrics for the models described previously is shown.

### Trend Extraction

**Figure 4:**
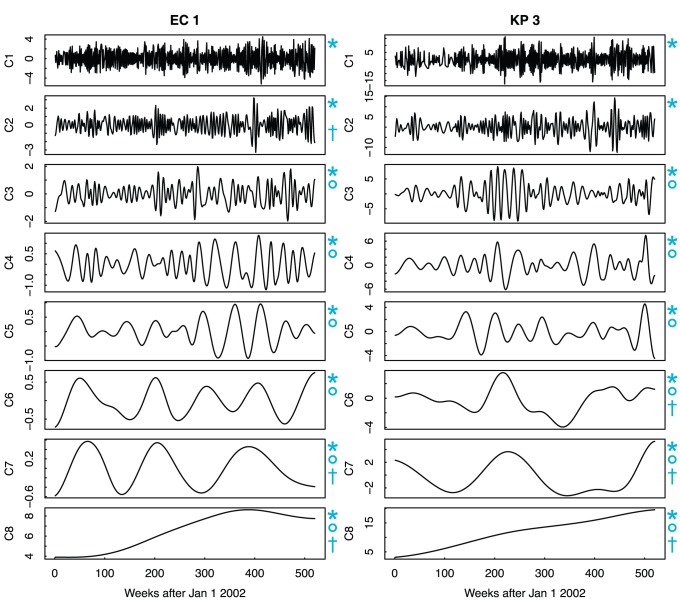
Result of the EMD technique applied to resistance trend extraction. In the example, EMD is used to decompose time series *EC 1* and *KP 3*. Components C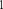 and C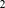 describe short-term resistance trends; components C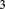 and C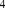 describe medium-term trends; and components C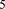 to C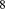 describe long-term trends. The residue of the decomposition process (C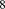) provides the underlying mean trend of the resistance signal. Components used in the *DECA, °*DECF* and ^†^ *DECS* models.

We have applied the EMD algorithm for extracting antibiotic resistance trends to the entire set of Table 1. The resulting components were used for trend analyses but also in the learning algorithm. A representative example of using EMD for antimicrobial resistance trend extraction is shown in Figure 4, where the time series *EC 1* and *KP 3* are decomposed into eight independent components (see Figure S2, S3, S4, S5 for the remaining list of time series decomposition). The first seven components (C_1_−C_7_) correspond to the IMFs and describe short-, medium- and long-term periodic trends. The component C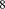 is the residue of the sifting process and represents the slowly varying mean resistance trend. The raw weekly resistance signal is equivalent to the sum of the eight components. The first component presents the highest frequency and as the component index increases, the frequency decreases. The same pattern is verified for the other time series and it is inherent to the EMD algorithm. The mean resistance trend is determined empirically and, for *EC 1*, it approximates a sigmoid shape. Research has shown that a sigmoid is the resistance pattern expected under constant selective pressure [13]. If the actual resistance dynamics of time series *EC 1* is indeed sigmoidal, then the resistance has reached its equilibrium and, from component C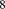, it becomes trivial to detect the resistance stabilization point, which happens to be around week 380. Considering that the resistance has started to increase around week 80, it took thus 5.8 years to reach the stabilization point. Similar rising period has been verified in other studies [13]. Differently, for *KP 3*, the resistance has not reached the stabilization point, maintaining a steady and linear rise.

#### Periodicity of resistance trends

**Figure 5:**
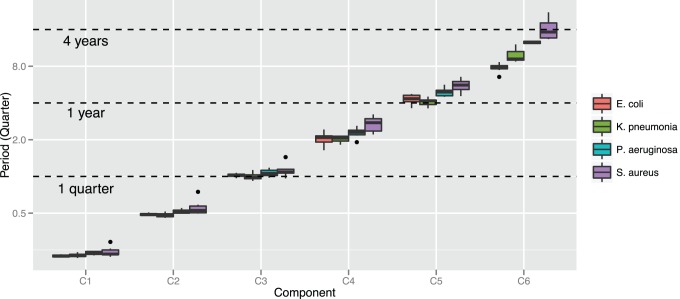
Oscillatory period in quarters of the resistance trend components stratified into pathogen groups. Components C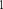 and C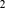 describe short-term variations in the resistance rates (median period 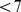 weeks); components C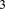 and C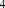 describe medium-term variations (median period between 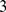 and 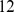 months); and components C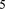 and C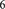 describe long-term changes (median period 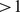 year). Notice how the period of the resistance trends are tightly associated to meaningful calendar cycles.

By counting the zero-crosses of the IMFs, we can extract the central period of oscillation of the resistance trend components. Figure 5 shows for the four groups of time series in Table 1 the results of applying this methodology. The oscillatory periods are given in weeks and are limited to the first six components. Components C_1_ and C_2_ have the smallest periods (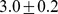 weeks and 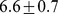 weeks respectively) and provide information on short-term trends. Components C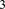 (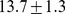 weeks) and C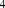 (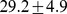 weeks) represent medium-term variations, with periodic trend oscillation varying between a quarter and a year long. Finally, components C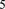 (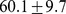 weeks) and C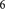 (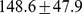 weeks) represent long-term trends, with periods longer than one year.

#### Correlation with resistance factors

Instead of seeking a direct relationship between time series, correlation can be computed using the intrinsic trend components extracted through the EMD technique [32]. In this case, the resistance process is seen as a sum of underlying physically meaningful factors that contribute independently to the increase and decrease of the overall resistance trend. To illustrate that, we use a temperature time series of the Geneva region as the external factor that may be associated to the resistance evolution at HUG. The temperature series comprises monthly sampled data for the same study period and is provided by the Federal Office of Meteorology and Climatology MeteoSwiss. The correlation is then computed between the components of temperature and those of the resistance time series (monthly aggregated). The results for the highest (positively or negatively) correlation coefficients are displayed in Table 2 (see Figure S6 for detailed correlation results). To avoid bias, we excluded residue-residue (C*_n_*_+1_) linkages, since by definition these are monotonic smoothed functions and therefore tend to present spurious correlation coefficients. The mean absolute correlation (0 to 1) between the original temperature and resistance time series is weak (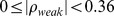) (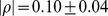). However, when we consider the particular EMD components of Table 2, the mean correlation becomes much stronger (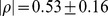). More specifically, time series *EC 2, KP 1, PA 3, SA 2* and *SA 6* contain components strongly (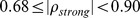) correlated with temperature components (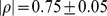), whereas *EC 1, EC 3, EC 4, EC 6, EC 7, KP 2, KP 4, KP 5, PA 4, PA 5, PA 6, SA 1, SA 3, SA 4* and *SA 5* show moderate (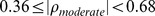) correlation (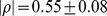), and *EC 5, KP 3, KP 6, KP 7, PA 1* and *PA 2* contain only weakly correlated components (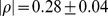).

**Table 2:** Correlation between resistance and temperature components.

Time	#Resistance	Component index		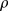	*P*-value	Signal
series	components	Temperature	Resistance			
EC 1	5	3	3	0.25	.007	0
EC 2	5	3	4	−0.79	<.001	1
EC 3	5	5	4	−0.47	<.001	1
EC 4	5	2	4	0.20	.03	0
EC 5	5	3	4	0.29	<.001	0
EC 6	5	3	4	0.47	<.001	0
EC 7	6	4	5	−0.53	<.001	1
KP 1	6	4	5	0.71	<.001	1
KP 2	6	4	4	0.32	<.001	1
KP 3	5	4	4	0.31	.001	1
KP 4	6	4	5	0.65	<.001	1
KP 5	6	4	5	0.54	<.001	1
KP 6	5	4	4	−0.27	.002	1
KP 7	6	5	4	0.34	<.001	1
PA 1	5	2	3	0.25	.006	1
PA 2	5	2	3	0.25	.006	1
PA 3	6	4	5	0.73	<.001	0
PA 4	6	3	3	0.49	<.001	0
PA 5	5	4	4	−0.58	<.001	1
PA 6	6	3	5	−0.45	<.001	1
SA 1	5	3	4	−0.65	<.001	1
SA 2	7	5	6	−0.82	<.001	1
SA 3	5	3	4	−0.55	<.001	1
SA 4	5	4	4	0.50	<.001	1
SA 5	6	3	4	−0.66	<.001	1
SA 6	5	4	4	−0.69	<.001	1

Results for the correlations with highest significance between components of monthly resistance and temperature time series. The column *Signal* indicates whether the associations are created from mutually statistically significant components (1) or from noisy components (0).

The quality of the above correlations can be further checked through a statistical significance test step. In this approach, we validate the linkages assuming that the components used in the correlation are either meaningful signals, in which case the linkage is valid, or noisy components, where the results should be discarded. For that, we recur to the statistical test proposed by Wu and Huang [26], by which components can be distinguished from a pure white noise series represented by the first IMF. If the linkages are created using mutually statistically significant components, then we consider that they are also significant. As for detecting the signal components in the *DECS* model, we use a 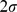 margin in the statistical test. The results are shown in the column *Signal* of Table 2. Applying the EMD algorithm to the monthly temperature and to the resistance time series yields 5 and median of 5 (interquartile range 5 to 6) components respectively, where components C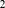, C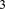, C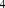 and C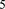 of temperature are statistically significant different from component C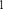. It turns out, under such a criterion, that all significant correlations but those for *EC 1, EC 4, EC 5, EC 6, PA 3* and *PA 4* time series contain at least one overlapping significant component with temperature, that is, they are bordering on the statistically significant at 95.4% confidence level. Thus, results for *EC 1, EC 2, EC 4, EC 6* and *PA 3* are discarded, leading to 20 out of 26 valid linkages and a still moderate level of correlation (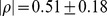).

### Resistance Forecasting

In the next sections, we show the results of the forecasting algorithm applied to test points varying from week 351 (September 2008) to 520 (December 2011) and trained on the respective 350 to 519 data points. Our first task is to determine the optimal size of the embedding dimension 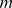. For that, the time series *EC 4, KP 2, PA 5* and *SA 5* are taken as the training set. We use this information in the final model evaluation, which excludes the aforementioned four time series from the training and test sets to avoid overfitting bias.

#### Embedding dimension

There were no statistical differences between the forecasts using any of the naïve methods and the method based on the fractal dimension 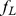 to determine the optimal embedding dimension 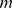 in the experiments with 1, 3 and 12 forecasting horizons. Nevertheless, similarly to the results obtained in [30], the dynamic method was able to adapt to the different time series and compute a well performing 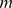, while keeping it small enough so as not to degrade the training and testing time. It resulted in an overall MAE of 5.57%, being the lowest MAE in 4 out of 12 tests – a result equivalent to the best naïve method (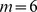). Thus, in the subsequent tests we employed the fractal dimension method in the *DECA*, *DECF* and *DECS* models to determine the size of the optimal embedding dimension *m*.

#### Forecasting models

**Figure 6:**
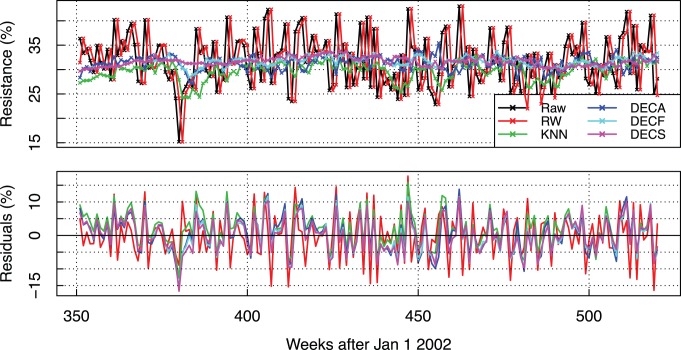
Example of forecasting results. *Top:* 1 week-ahead forecasting results for times series *EC 7*. *Bottom:* Respective forecasting residuals.

In Figure 6, we display a representative example of resistance forecasting and its respective residual error. In the top panel, the actual resistance rate is displayed in black whereas the 1 week-ahead forecasts for the *RW* (random walk), *KNN* (k-NN without trend decomposition), *DECA*, *DECF* and *DECS* models are shown in red, green, dark blue, light blue and purple, respectively (see Figure S7 for forecasts of the remaining time series). The predictions of the baseline model follow the signal but are always lagged by one data point (one week in this case). Thus, this model presents the largest absolute residuals, caused especially by stochastic zigzag variations in the resistance time series. The *KNN* and *DECA* models, despite using the full signal spectrum, are not able to capture high frequency changes either. They forecast medium-term trends but without accuracy. Finally, the *DECF* and *DECS* models learn essentially the underlying mean trend.

**Figure 7:**
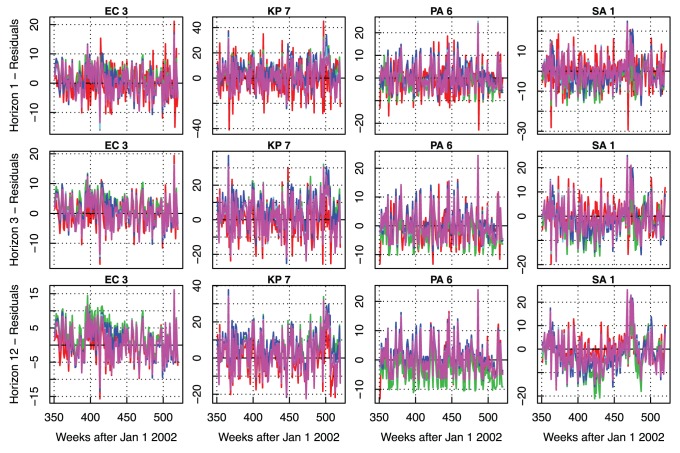
Example of forecasting residuals for 1, 3 and 12 week-ahead horizons. Results for four representative time series of each pathogen group **–** *EC 3*, *KP 7*, *PA 6* and *SA 1*. *RW*: red; *KNN*: green; *DECA*: dark blue; *DECF*: light blue; *DECS*: purple.

The prediction patterns are consistent as the forecasting horizons and time series changes. For example, in Figure 7, the residual errors of the *EC 3, KP 7, PA 6* and *SA 1* time series are displayed for 1, 3 and 12 forecasting horizons using the same color schema of Figure 6. The row panels correspond to forecasting horizons and the column panels correspond to the different time series. Similarly to Figure 6, given that each of the models uses different forecasting algorithms, none is still able to forecast the high-frequency trend components. The short-term spikes appear in all the residual results as we walk along the columns. Furthermore, they do not increase significantly with the forecasting horizon, being of the same order of magnitude. Finally, the column residuals for a specific model are highly correlated (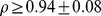).

The MAE and RMSE prediction errors for 1 week-ahead horizons generated by the various forecasting models are displayed in Table 3. Overall, the models that employ decomposition of the time series and filter out noisy components, that is, *DECF* and *DECS*, improve significantly the forecasting accuracy over the other models for both error measures (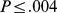). The *DECS* model performs slightly better than the *DECF* model for both MAE and RMSE metrics. However, their difference in forecasting accuracy is not statistically significant (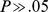).

**Table 3:** Accuracy of 1 week-ahead forecasting **–** mean absolute (MAE) and root mean squared (RMSE) errors.

		Time series										
Error	Method	EC 1	EC 2	EC 3	EC 5	EC 6	EC 7	PA 1	PA 2	PA 3	PA 4	PA 6
	RW	3.15	5.78	4.42	4.56	5.19	5.64	4.62	6.13	3.46	4.10	5.46
	KNN	2.52	4.74	4.00	3.72	4.06	4.66	3.95	4.86	**3.01**	**3.70**	**4.59**
MAE	DECA	2.54	4.68	3.65	3.56	4.08	4.50	4.02	4.74	**3.01**	**3.84**	**4.68**
	DECF	**2.50**	**4.46**	**3.67**	**3.51**	**3.98**	**4.32**	**3.87**	**4.67**	**3.06**	**3.64**	**4.23**
	DECS	**2.50**	**4.50**	**3.64**	**3.37**	**3.94**	**4.37**	**3.89**	**4.73**	**3.03**	**3.37**	**4.29**
	RW	3.79	7.35	5.64	5.76	6.62	7.07	6.02	7.71	4.67	5.49	7.07
	KNN	3.07	5.85	4.93	4.79	5.36	5.70	4.85	6.24	3.60	4.47	5.76
RMSE	DECA	3.04	5.75	4.73	4.68	5.35	5.45	5.01	6.19	3.74	4.75	5.95
	DECF	2.94	5.51	4.65	4.60	**5.15**	**5.28**	**4.76**	**6.05**	**3.68**	**4.47**	**5.42**
	DECS	**2.93**	**5.47**	**4.59**	**4.36**	**5.17**	**5.30**	**4.84**	**6.08**	**3.57**	**4.19**	**5.48**
		Time series										
**Error**	**Method**	**KP 1**	**KP 3**	**KP 4**	**KP 5**	**KP 6**	**KP 7**	**SA 1**	**SA 2**	**SA 3**	**SA 4**	**SA 6**
	RW	6.08	8.83	8.85	8.18	5.69	10.97	6.51	4.38	7.28	7.44	1.40
	KNN	**5.14**	**7.41**	**7.46**	**6.69**	**5.35**	**8.89**	**5.82**	**3.88**	**6.50**	**6.86**	**1.27**
MAE	DECA	5.20	8.10	7.95	7.04	5.80	9.24	5.66	3.95	6.49	6.68	**1.20**
	DECF	5.19	7.50	7.59	**6.05**	**5.59**	**8.45**	**5.28**	**3.80**	**5.99**	**6.25**	**1.20**
	DECS	5.35	7.67	**7.33**	**6.41**	**5.53**	**8.19**	**5.14**	**3.87**	**6.06**	**6.20**	**1.24**
	RW	7.89	11.66	11.70	9.98	7.82	13.85	8.28	5.73	9.22	9.53	2.08
	KNN	**6.26**	**9.74**	**9.78**	**8.30**	**7.11**	**11.05**	**7.31**	**4.94**	**8.24**	**8.55**	**1.53**
RMSE	DECA	6.67	10.54	10.44	8.62	7.65	11.39	7.27	5.14	8.20	8.49	1.49
	DECF	6.51	**9.67**	**9.76**	**7.80**	**7.26**	**10.58**	**6.83**	**4.78**	**7.59**	**7.82**	**1.48**
	DECS	6.58	9.99	**9.55**	**8.07**	**7.22**	**10.20**	**6.54**	**4.85**	**7.69**	**7.80**	**1.49**

Results for the best forecasting performance are displayed in bold. Overall, the *DECF* and *DECS* methods have the smallest prediction errors, outperforming the other methods for all but time series *PA 3*, *KP1*, *KP3* and *KP 6* when we consider the MAE metric.

**Figure 8:**
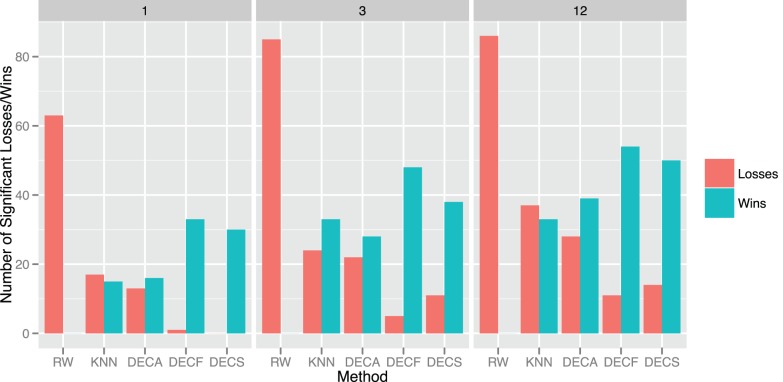
Statistical comparison of the different forecasting methods using the mean absolute error (MAE) for 1, 3 and 12 week-ahead horizons. *Wins*: frequency that a method significantly outperforms other methods. *Losses*: frequency that a method is significantly outperformed by other methods.

To compare the forecasting models at 1, 3 and 12 forecasting horizons, we perform a statistical significance test of accuracy using the MAE values. The results are displayed in Figure 8, where the frequency that a given model significantly outperforms the other models (wins) is shown in green and the frequency that a model is outperformed by the other models (losses) is shown in red. As we can see, the *DECS* and *DECF* models have the best performance in all forecasting horizons, that is, they have the highest number of wins (*DECF*: 135, *DECS*: 118) and smallest number of losses (*DECF*: 17, *DECS*: 25). As the horizon increases, the power of the models that use decomposition becomes more evident. Particularly, at the 12 week-ahead horizon they outperform both the *RW* and *KNN* models. Finally, all the k-NN-based models improve upon the *RW* baseline model.

**Figure 9:**
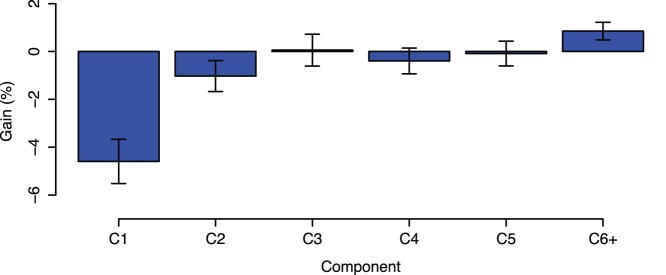
Contribution of the individual components to the forecasting model. While low frequency components improve the prediction accuracy, high frequency components worsen the model.

The impact of the individual components (C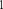, C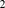, …, C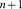) in the *DECA*, *DECF* and *DECS* forecasting models can be assessed through a systematic evaluation of the forecasting errors. In this approach, we compare the relative gain of incrementally including higher frequency components in the model. If there is a positive gain, that is, the forecasting accuracy increases, then the component adds value to the model. Otherwise, it is irrelevant or may even degrade the quality of the predictions. The results of performance gain in terms of MAE for 1 week-ahead forecasts are shown in Figure 9. The model containing only the component C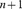 (residue) is taken as the reference. Then, the relative gain is computed for the other models: C1 includes all components (equivalent to *DECA*), C2 includes all components but C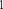, C3 includes all but C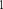 and C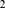 (equivalent to *DECF* – notice from Figure 5 that periods of components C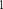 and C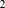 are smaller than 10 weeks while periods of components C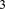 to C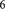 are greater than 10 weeks) and so forth up to C6+, which includes components C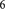 to C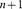 (at any testing point, the resistance time series had at least seven components). We can distinguish three segments: C1 and C2 degrade the forecasting accuracy (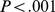 and 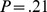) by 4.6% and 1.0% respectively, C3 to C5 do not impact significantly in the model (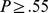), with gains varying between −0.4% to 0.1%, and C6+, which improves the overall model (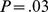) by 0.9%.

## Discussion

In the present study, we developed a two-stage model for temporal analyses of antibiotic resistance data using trend extraction and forecasting methods applied to up-to-date and short-term trends. Our model was validated in a large scale data set spanning a decade of weekly aggregated resistance time series. The use of the EMD algorithm for trend extraction was effective not only to obtain the resistance trends, but also to provide insight into the periodicity of the resistances and into the level of correlation with external variables. The machine learning forecasting models supported by the k-nearest embedding vectors produced results with good accuracy, statistically outperforming the baseline approaches. The decomposition of the raw signal and exclusion of the noisy components had a positive impact to reduce the forecasting error. Finally, both trend extraction and forecasting methods proved to be robust, adapting to time series of different resistance dynamics.

We focused on short-term trends because they are able to capture more efficiently the resistance dynamics within a given clinical setting. Especially in cases of resistance outbreaks, monthly and yearly resistance trends cannot spot readily changes in the mean rates. For example, in the vancomycin-resistance *Enterococcus* outbreak experienced at Princess Alexandra Hospital, Brisbane, Australia in 1999, in approximately 10 weeks the number of resistant prevalence cases increased 14 fold, even if an abnormal prevalence rate had already been detected in the first week of the outbreak [33]. Thus, effective biosurveillance systems should apply timely trend analysis methods to avoid further spreading of resistance strains.

### Trend Extraction

We have explored the EMD algorithm to extract antibiotic resistance trends from weekly aggregated time series. Traditionally, infectious disease specialists use monthly and yearly resistance data and statistical tests to assess resistance trends [34]. In comparison, the methodology introduced here provides improved insight into the dynamics of resistance than the simple detection of upward/downward trends. First, it is able to extract medium- and long-term variations in the resistance rate but it can also capture short-term changes through the IMF components, which are neglected in trend detection analyses. Second, some components of the resistance signal, especially those with high frequency, could be further used in biosurveillance systems as an early warning of emerging resistance, particularly if using data from high antibiotic pressure units, like intensive care. When combined with forecasts, which serve as the reference signal, the variation of components like C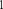 and C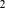 may be applied to differentiate between a normal fluctuation of resistant strains and the start of an outbreak, determined when the component’s amplitude extrapolates the 95% variance interval [35]. Third, infectious disease specialists can use the methodology to determine periodicity and cycles within resistance trends from the decomposed components and may adopt infection control interventions accordingly. For instance, component C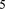 in Figure 5 has a period slightly longer than one year, which could be related, for example, to warm and cold seasons or, more precisely, to high winter peaks of antibiotic use as verified in the study presented in [36]. Therefore, the EMD methodology may serve as a complementary tool for the analysis of short-term antibiotic resistance data, and eventually, to help controlling long-term resistance trends as a result of prompt preventive measures.

The components extracted using the EMD technique may be further applied to improve correlation analyses between antibiotic resistance evolution and variations in other clinical, societal and environmental factors, such as duration of treatment, infection control measures, antibiotic consumption and weather [17], [36]. IMFs are orthogonal functions to one another and can thus be interpreted as independent factors. Moreover, they may sometimes represent biologically meaningful events in the resistance process [26], . These events might not always be explicit in the raw signal, sometimes undermining any attempt to associate their effect upon changes in resistance when using only the raw time series. For example, as demonstrated in the correlation between resistance and temperature time series, while the raw time series showed week correlation, some EMD components had moderate and high correlation coefficients. To further illustrate that, imagine that in a given clinical setting resistance has increased 1% due to antibiotic misuse and decreased 1% due to better hand hygiene practices, resulting in no changes in the raw resistance signal (see around week 300 of time series *EC 1* in Figure 4 for a concrete example of effects acting in opposite direction, where the wave forms are negative for components C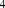 and C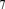 and positive for C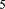 and C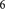). Hence, the attempt to correlate one of these factors – antibiotic consumption or hand hygiene practices – with raw resistance changes are likely not going to yield any conclusive results. On the other hand, if antibiotic consumption and hand hygiene have independent effect upon resistance, that is, they have different timing (phase and frequency) or power (amplitude), their effect shall be captured in the decomposition process, allowing thus a more fine grained correlation analysis.

### Resistance Forecasting

We have developed a novel machine learning method to forecast antibiotic resistance trends based on the k-nearest embedding vectors. The algorithm showed relatively good forecasting accuracy for short-term trends, outperforming baseline machine learning benchmarks but also other enhanced methods, such as the k-NN. The method is supported by the delay coordinate embedding theorem, a technique derived from the studies of chaos to model deterministic nonlinear time series, and by the k-NN framework to project observed resistance events in embedded spaces into the future dimension. From our experiments, we identified that decomposing the raw signal to enhance the features of the training data and excluding high frequency components from the learning set boosts the performance of the forecaster. This reinforces our hypothesis that some components of the resistance signals are derived from a pure random process. Hence, they cannot be learned by and degrade the quality of the learning algorithm. As further demonstrated in the systematic analyses of the individual components, high frequency IMFs, in particular C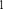 and C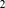, worsen the power of the algorithm. Therefore, they should be filtered out from the antibiotic resistance forecasting model to enhance its accuracy. These findings may imply that short-term aggregated resistance time series contain significant stochastic components and thus cannot be learned and predicted precisely using only past data.

Our results suggests that the k-nearest embedding vector model could be eventually used to improve clinical decision support systems for antibiotic prescribing, giving more accurate information on the current resistance dynamics than the latest resistance statistics when there are delays of a week or more in the resistance numbers. As shown in Figure 6, the forecasts provided by the naïve method, which was used as the baseline benchmark, are delayed by one week (notice the one-step forward shift between the red and black lines). As such, they are equivalent to the latest resistance data points, or 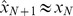, which are obtained in phenotypic antibiograms, in the best case, from samples extracted two or three days in the past. Since the models that use decomposition significantly improves upon the naïve method, by consequence, they also provide better evidence to empirical therapy and outbreak detection models than methods that use the latest resistance rate information when actual results are delayed by at least one data point.

### Limitations

This study used data aggregated from several wards. Thus, analyses restricted to a specific ward may lead to different outcomes. Nevertheless, as the methodology is data-independent, we believe it can be readily applied to such cases. Moreover, it was limited to time series of pathogens that present some level of resistance to the respective antibiotics. Sequences showing bursting patterns [38], as verified at the beginning of the resistance development process, were not tested and, from the forecasting results, it is unlikely that our model will be able to forecast bursts. Finally, we have not investigated the effect of irregular time series, that is, those containing null values. The time series of the study describe resistance information of bacteria with high prevalence rate, having at least one positive culture followed by an antibiogram per week.

### Conclusions

This paper presents a two-stage methodology for analyses of short-term antibiotic resistance trends. Using a large microbiology data set, we have developed a robust, fully data-driven methodology for trend extraction and forecasting of resistance time series. Our method, with decomposed resistance trends and appropriately selected model components, added valuable insight into the dynamics of the resistance time series and significantly outperformed the baseline forecasting algorithms. Hence, the method could potentially be used to improve outbreak detection and biosurveillance models within clinical settings. Moreover, since the trend extraction and forecasting methodologies do not assume any underlying model for the data set, it could be generalized to other time varying clinical events. Future research could be aimed at investigating the correlation of other time dependent clinical factors, such as antibiotic consumption, with decomposed resistance trends. Finally, the forecasting methodology could be combined with burst detection models to improve the prediction accuracy.

## Supporting Information

Figure S1 **Resistance time series for the test period.**
(PDF)

Figure S2 **Decomposition of the** ***E. coli*** **time series using the EMD method.** Components used in the *DECA, °*DECF* and ^†^ *DECS* models.
(PDF)

Figure S3 **Decomposition of the** ***K. pneumonia*** **time series using the EMD method.** Components used in the *DECA, °*DECF* and ^†^ *DECS* models.
(PDF)

Figure S4 **Decomposition of the** ***P. aeruginosa*** **time series using the EMD method.** Components used in the *DECA, °*DECF* and ^†^ *DECS* models.
(PDF)

Figure S5 **Decomposition of the** ***S. aureus*** **time series using the EMD method.** Components used in the *DECA, °*DECF* and ^†^ *DECS* models.
(PDF)

Figure S6
**Correlation between temperature and resistance components.** °,*components mutually statistically significant different from noise; ° correlation not significant (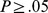); *correlation significant (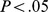).(PDF)

Figure S7 **Results for 1-weak ahead forecasts.** Raw signal: black; *RW*: red; *KNN*: green; *DECA*: dark blue; *DECF*: light blue; *DECS*: purple.
(PDF)
