# Supplementary figures and images for: Empirical Mode Decomposition and k-Nearest Embedding Vectors for Timely Analyses of Antibiotic Resistance Trends
Source: PLoS One. 2013 Apr 25;8(4):e61180. doi: 10.1371/journal.pone.0061180 (PMC3636283; doi:10.1371/journal.pone.0061180)

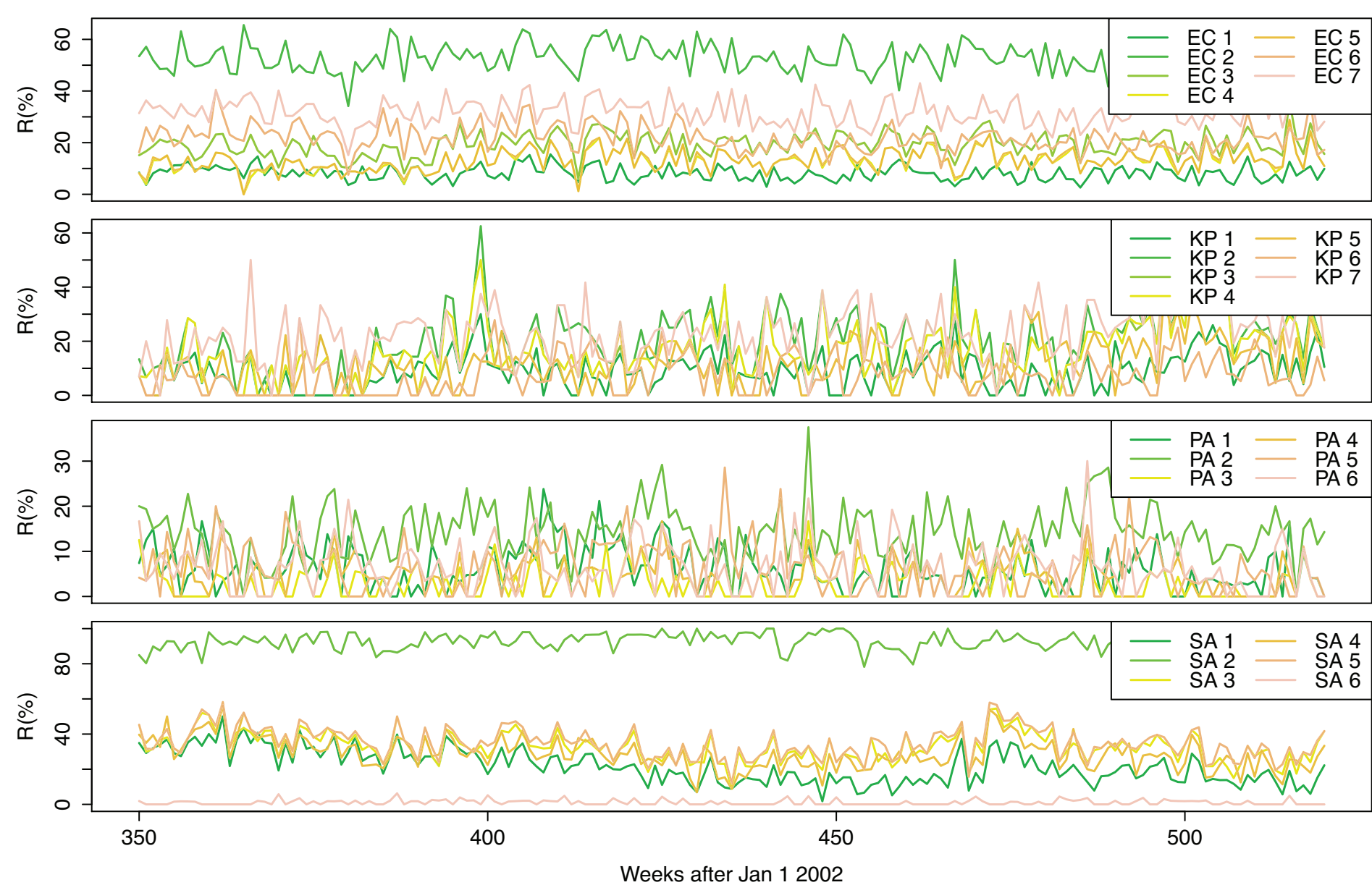

Supplement: Figure S1 — Resistance time series for the test period. (PDF) [file pone.0061180.s001.pdf]

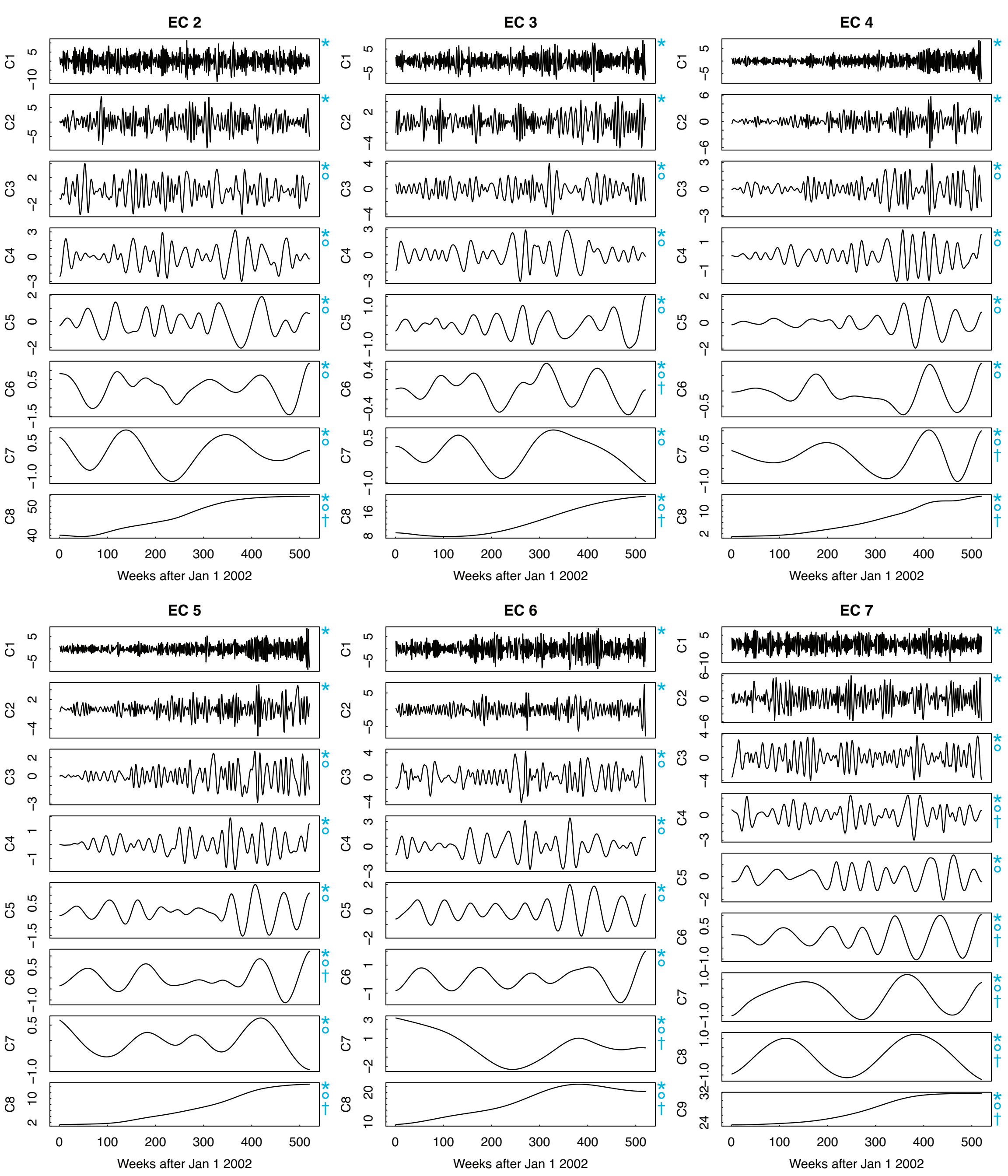

Supplement: Figure S2 — Decomposition of the E. coli time series using the EMD method. Components used in the *DECA, °DECF and † DECS models. (PDF) [file pone.0061180.s002.pdf]

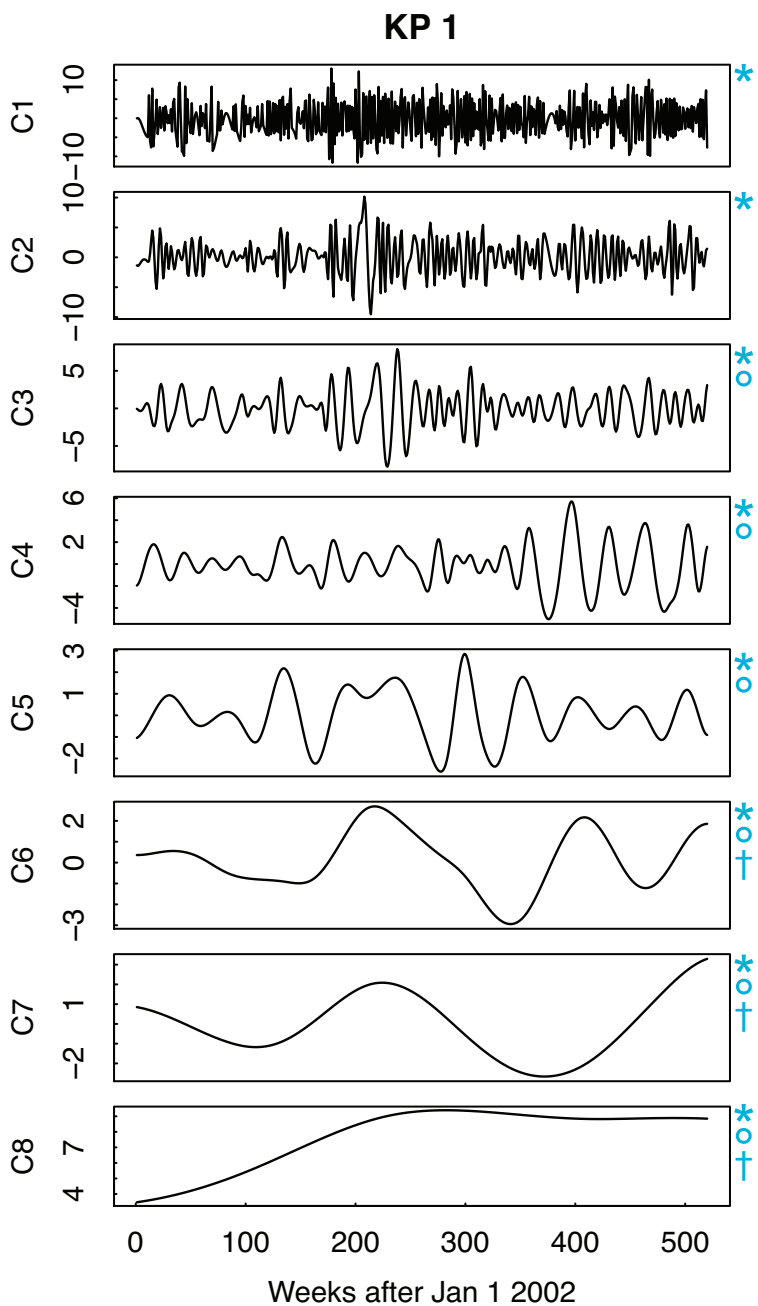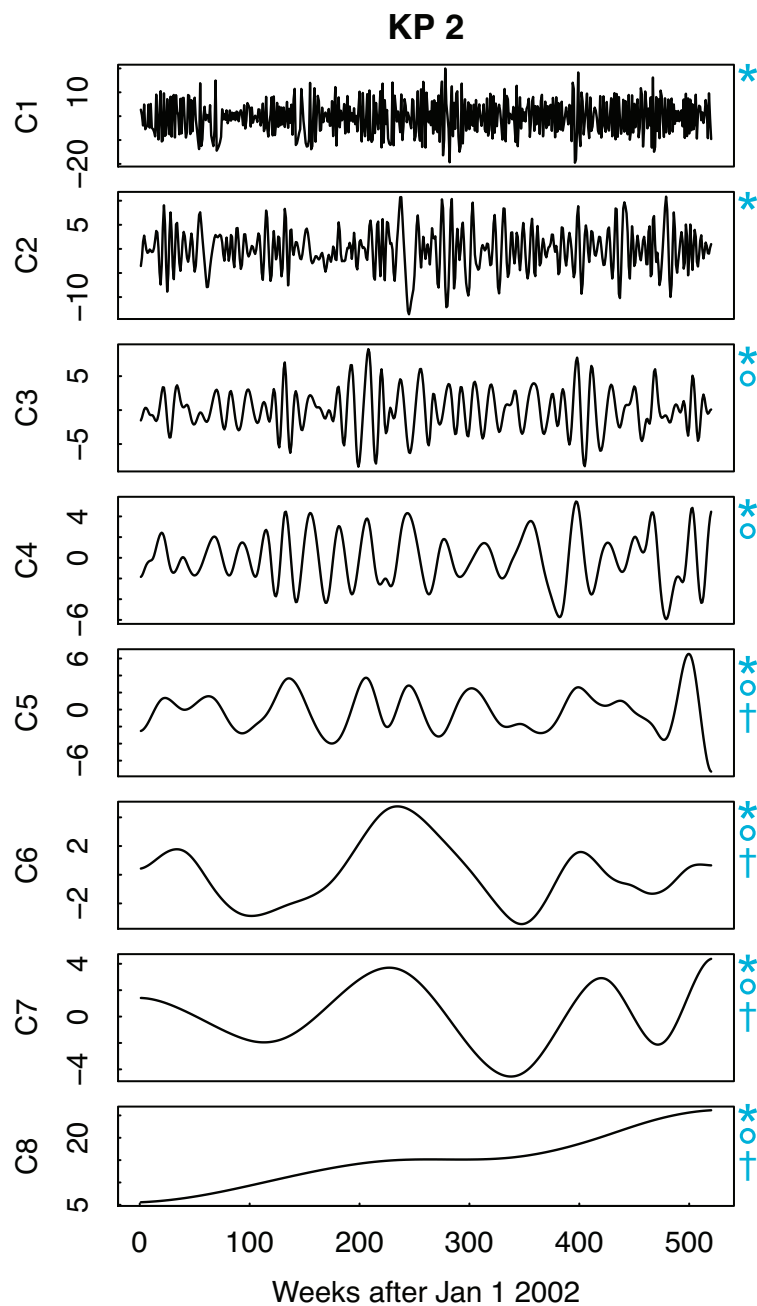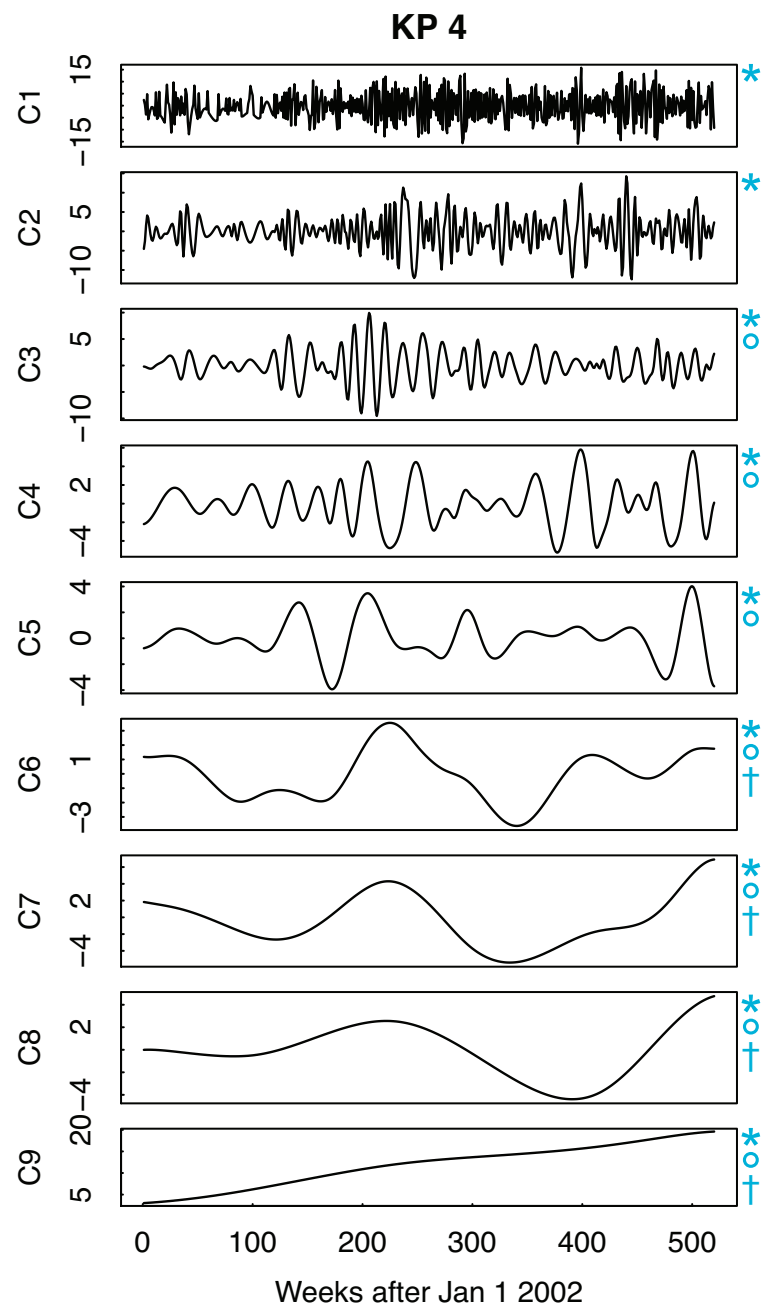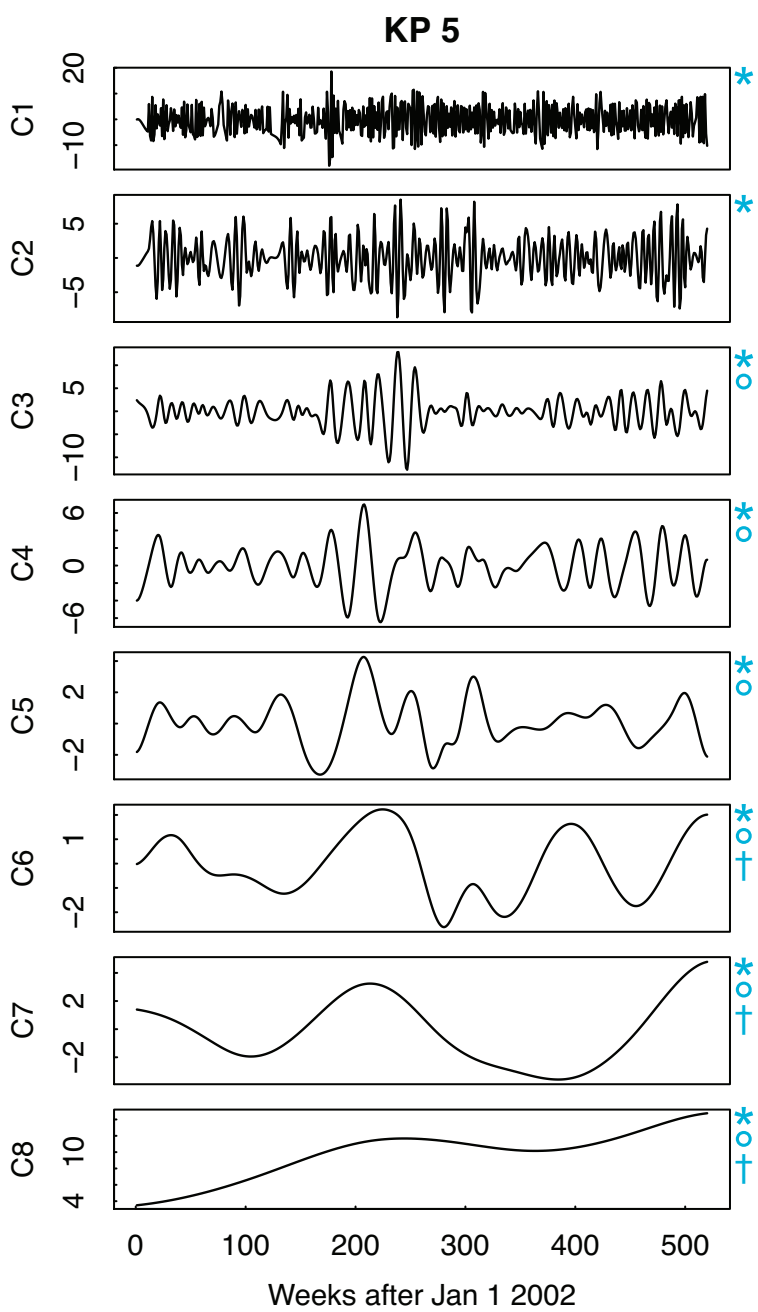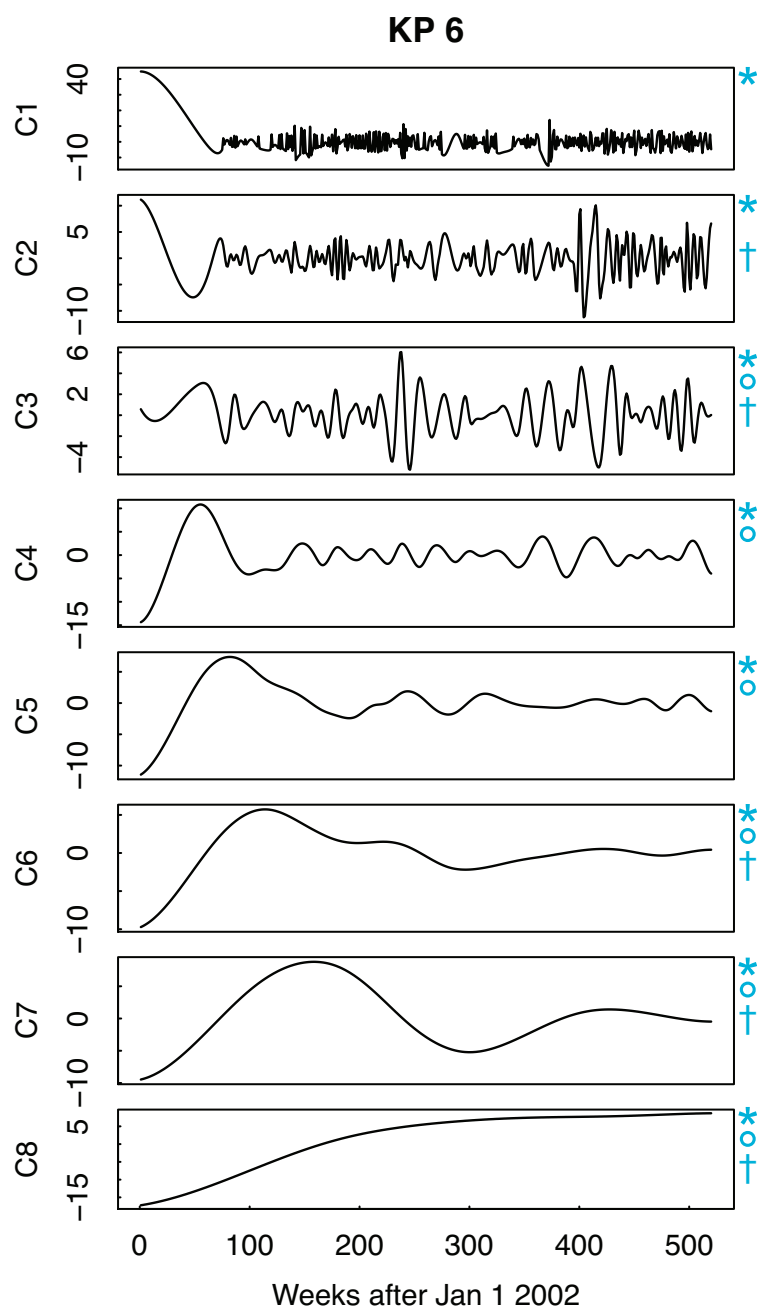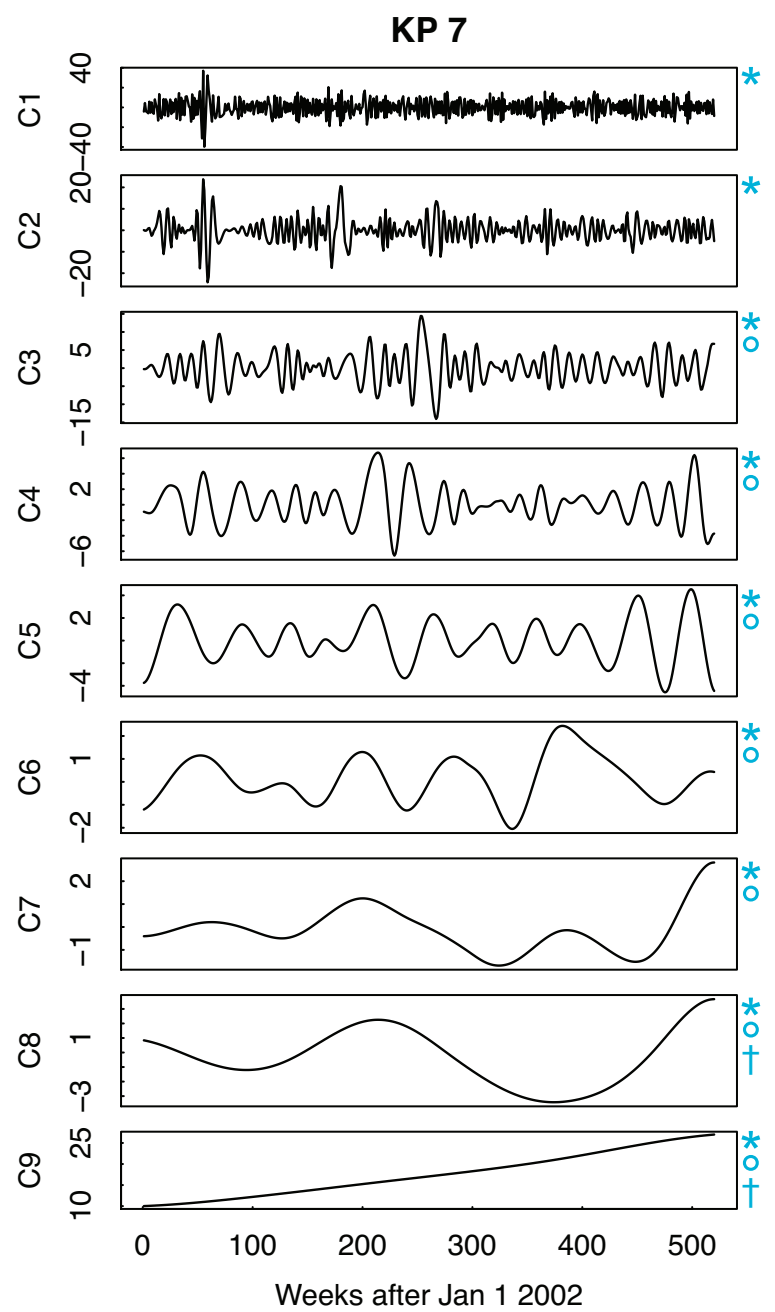

Supplement: Figure S3 — Decomposition of the K. pneumonia time series using the EMD method. Components used in the *DECA, °DECF and † DECS models. (PDF) [file pone.0061180.s003.pdf]

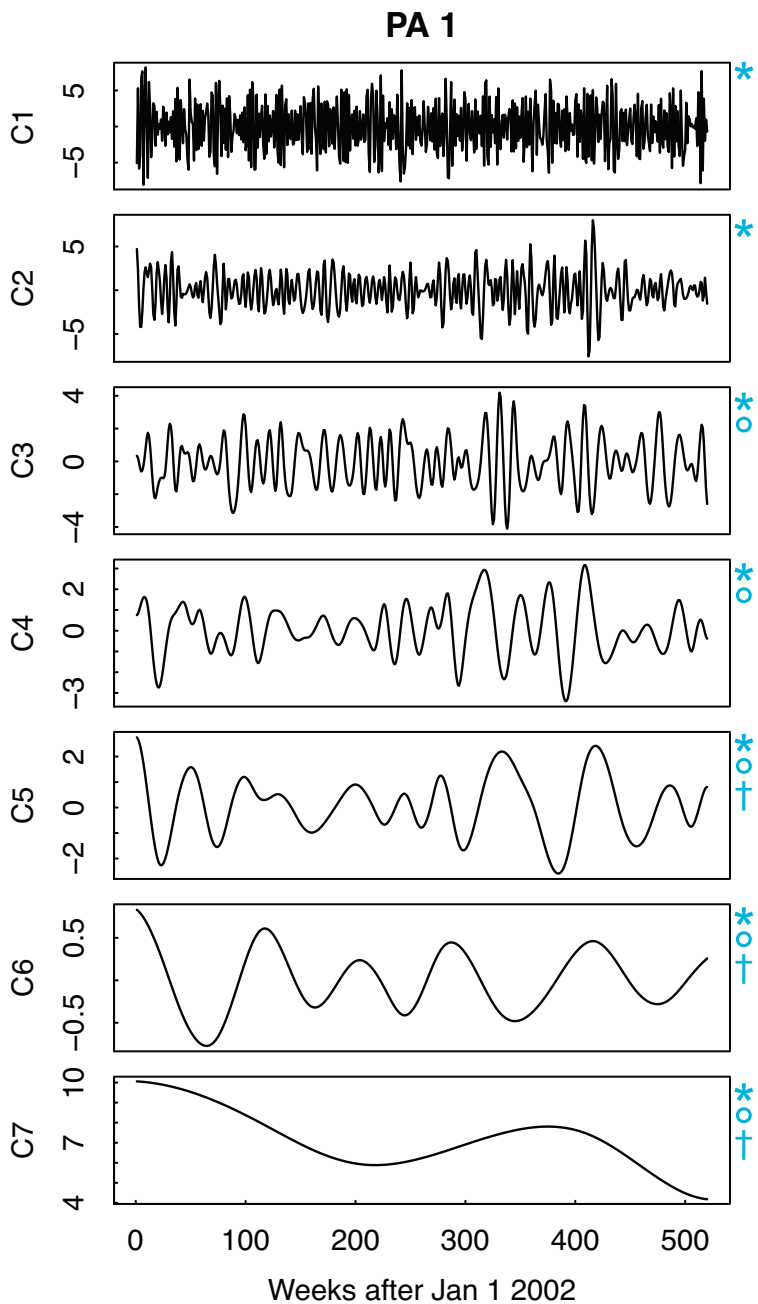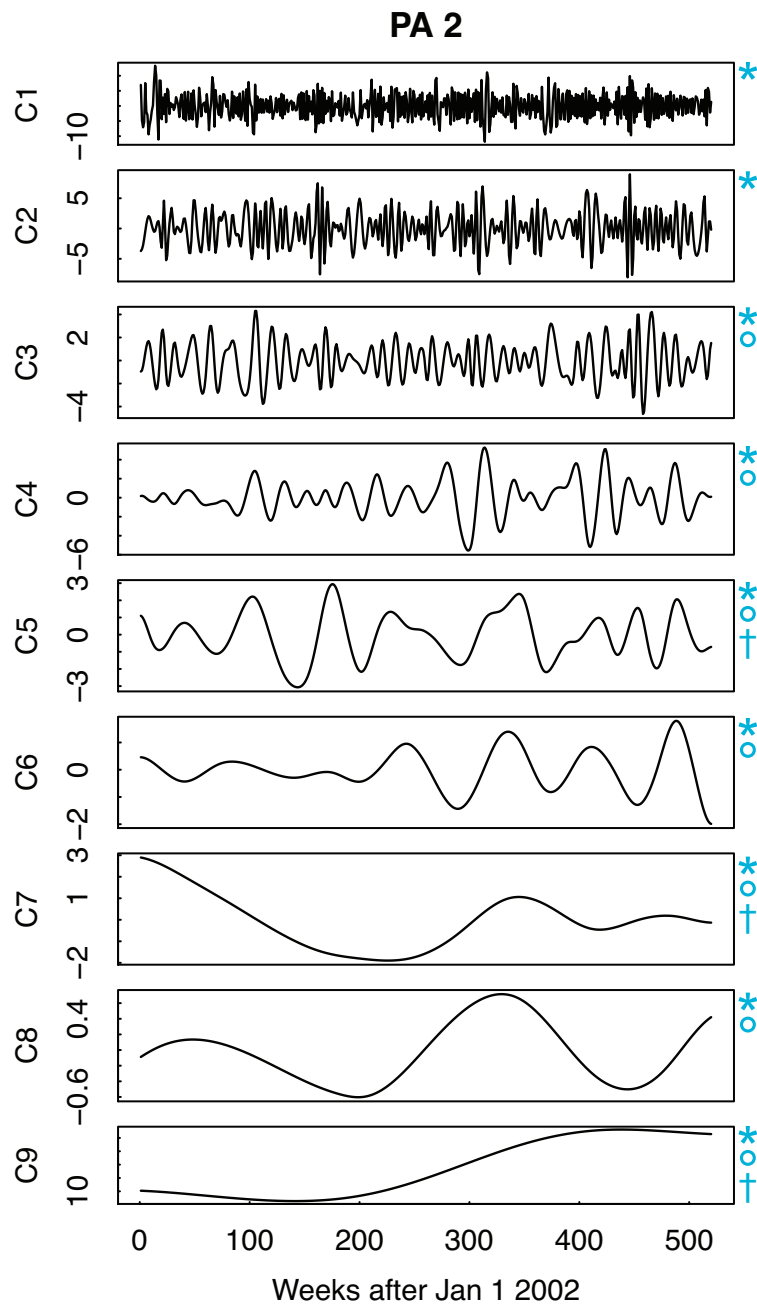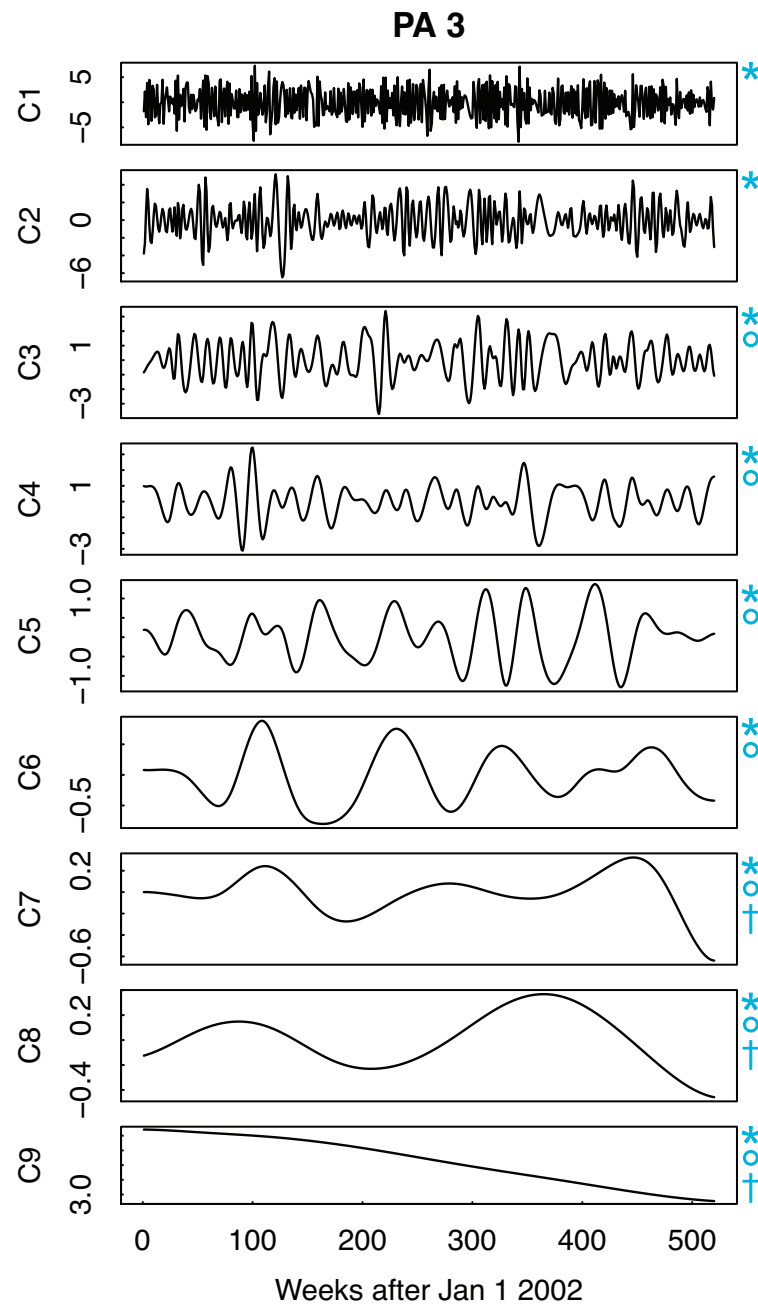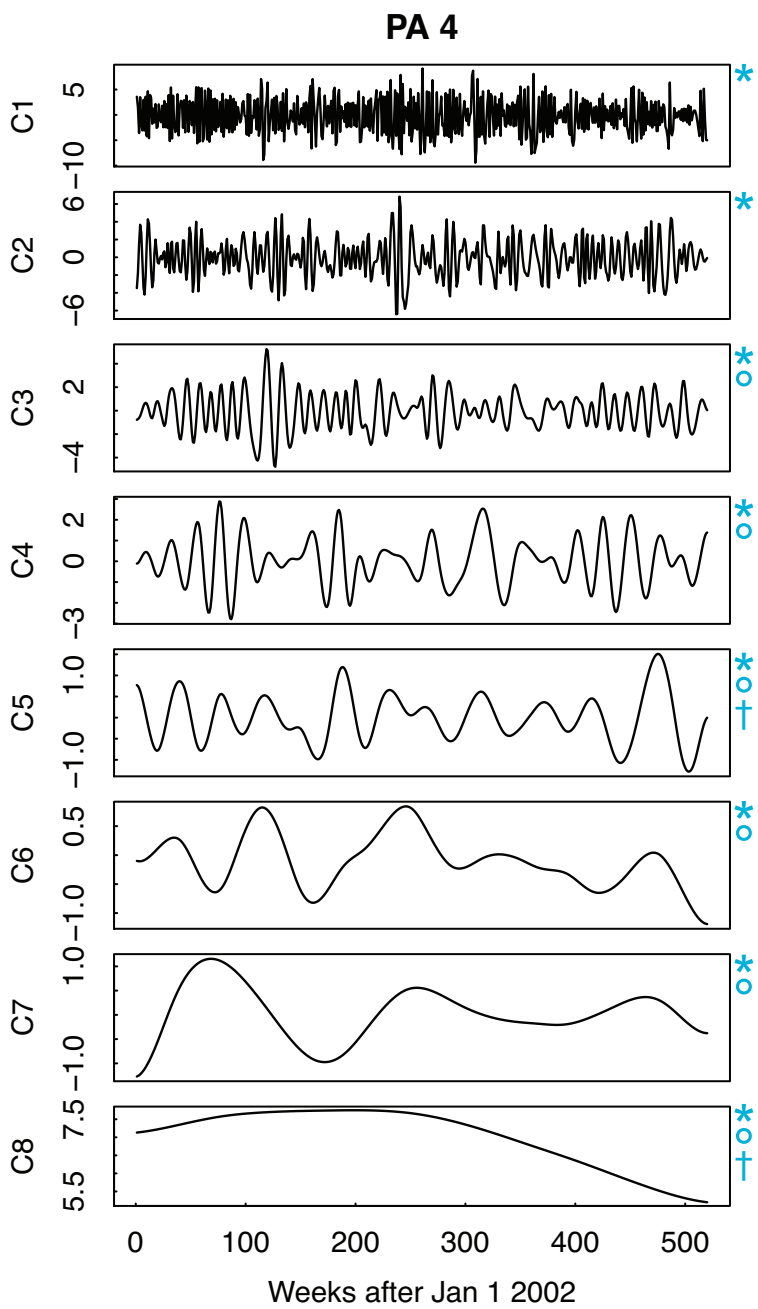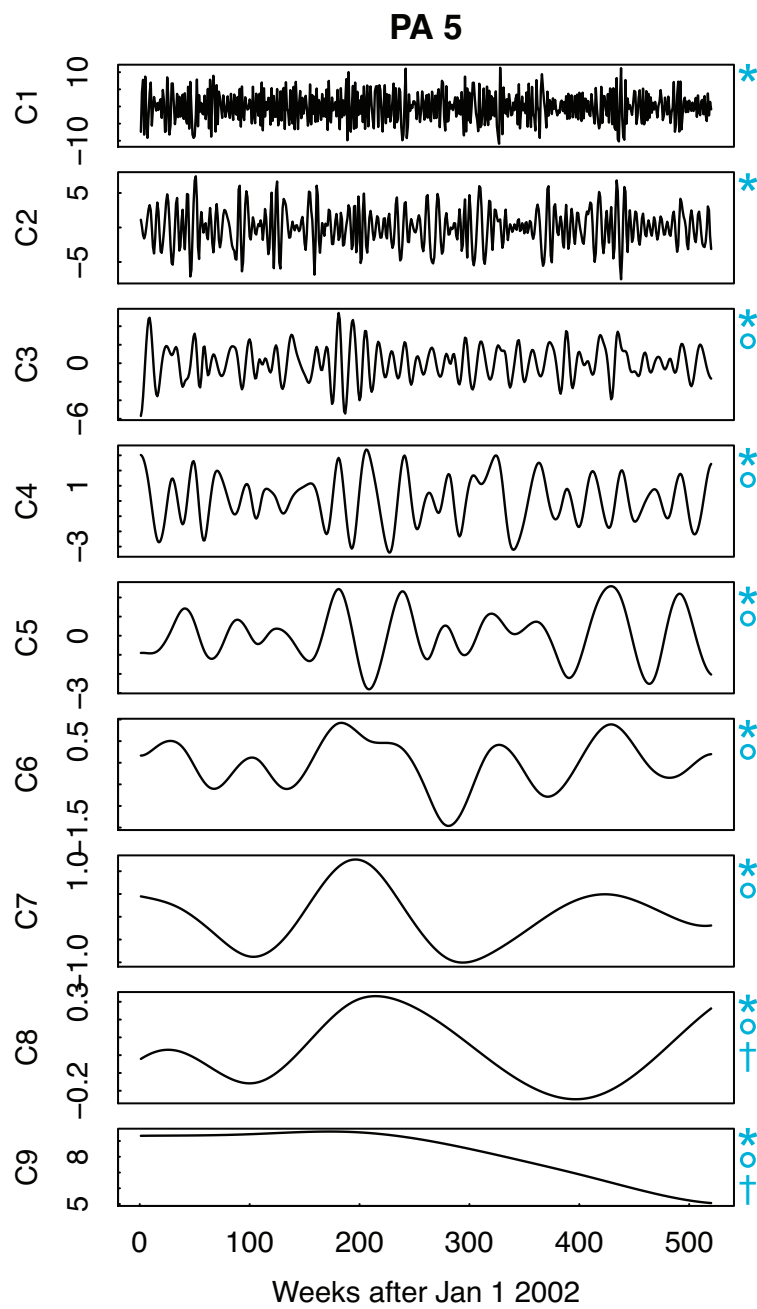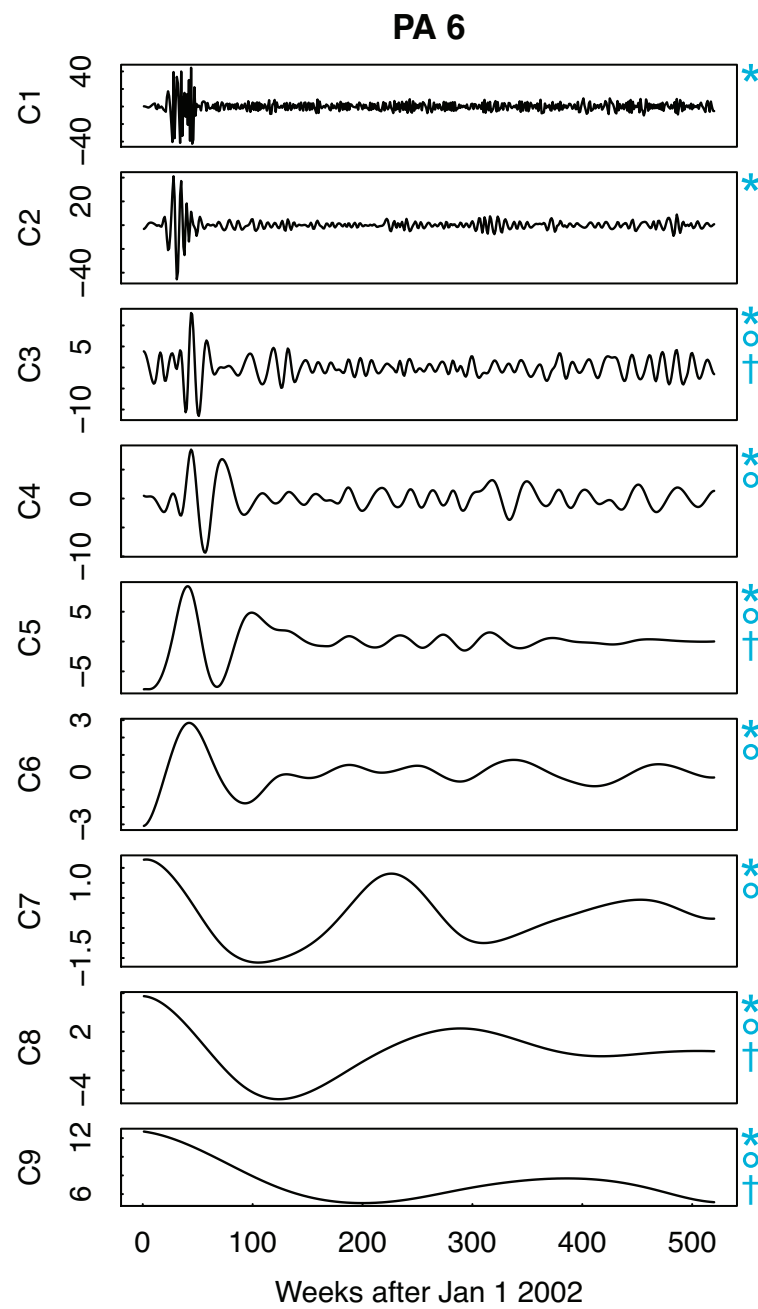

Supplement: Figure S4 — Decomposition of the P. aeruginosa time series using the EMD method. Components used in the *DECA, °DECF and † DECS models. (PDF) [file pone.0061180.s004.pdf]

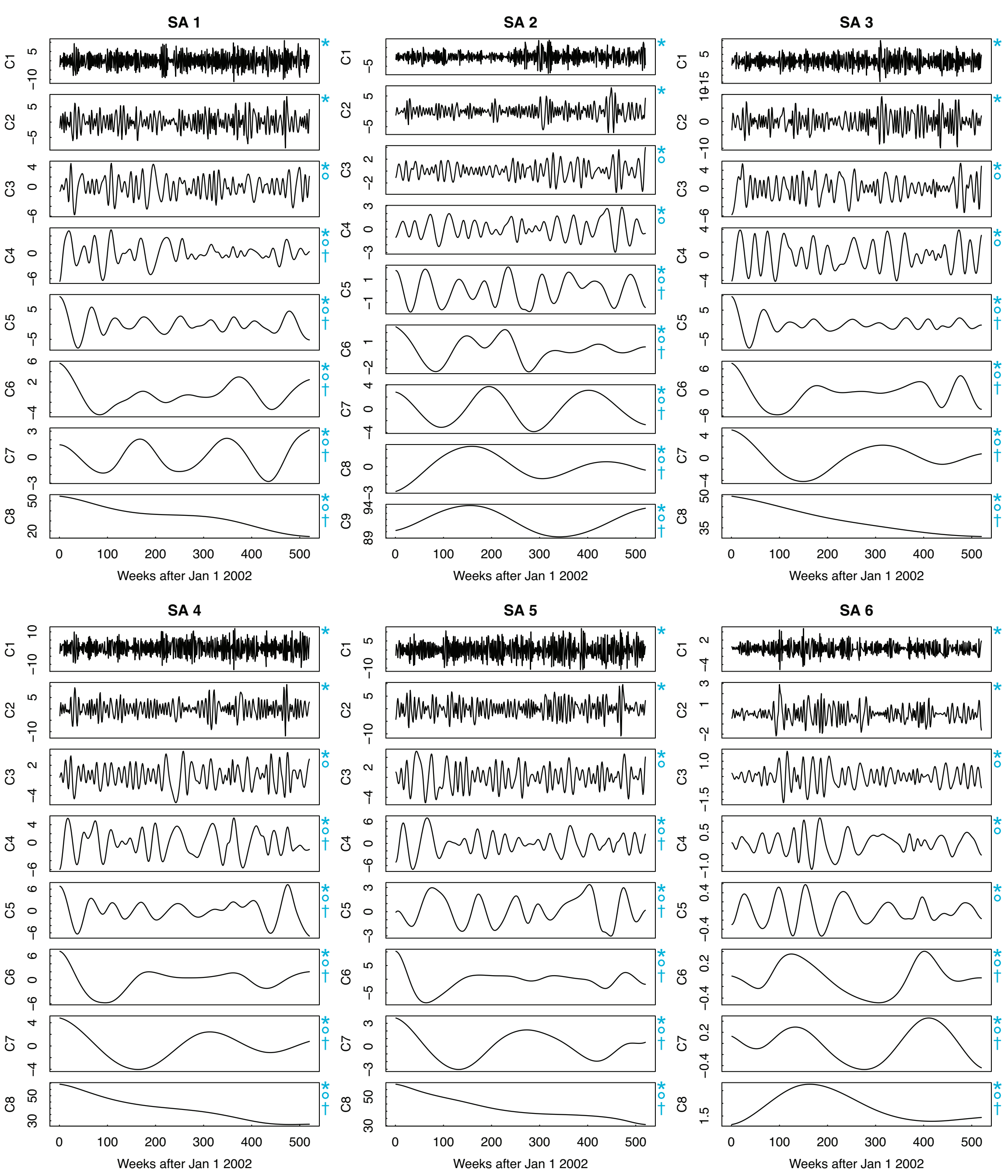

Supplement: Figure S5 — Decomposition of the S. aureus time series using the EMD method. Components used in the *DECA, °DECF and † DECS models. (PDF) [file pone.0061180.s005.pdf]

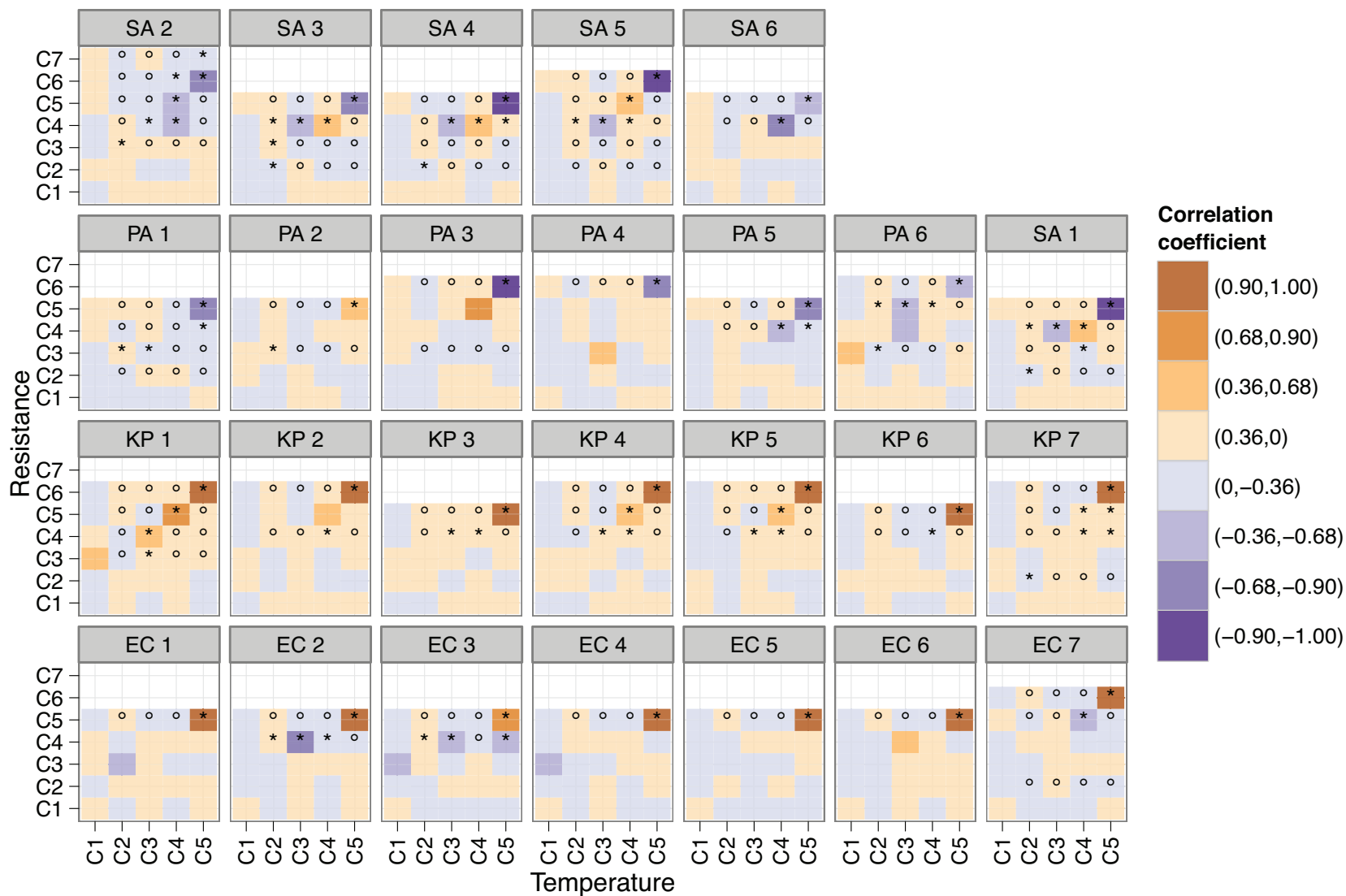

Supplement: Figure S6 — Correlation between temperature and resistance components. °,*components mutually statistically significant different from noise; ° correlation not significant (); *correlation significant (). (PDF) [file pone.0061180.s006.pdf]

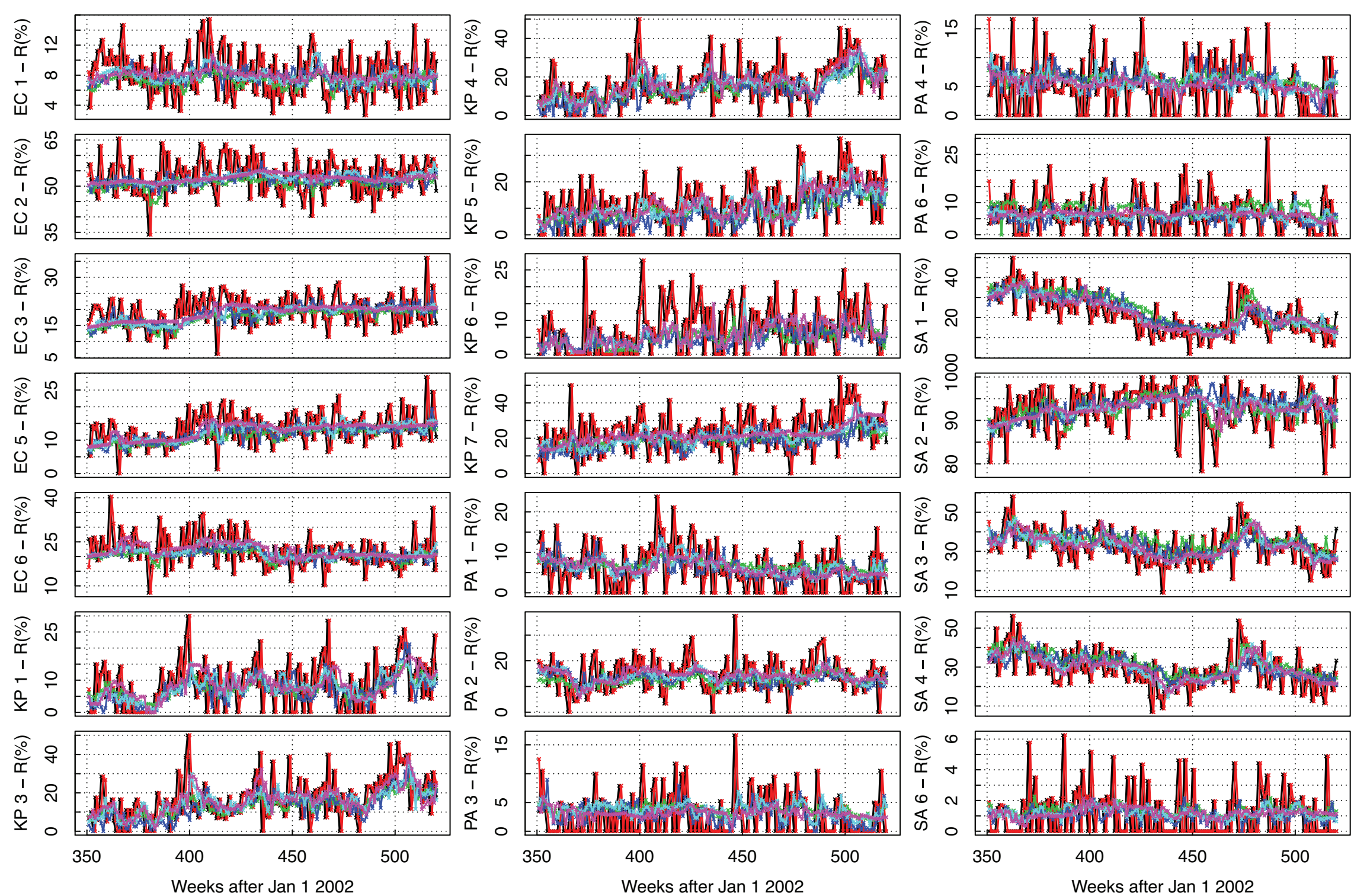

Supplement: Figure S7 — Results for 1-weak ahead forecasts. Raw signal: black; RW: red; KNN: green; DECA: dark blue; DECF: light blue; DECS: purple. (PDF) [file pone.0061180.s007.pdf]
